# Robust Adaptive Control of Fully Constrained Cable-Driven Serial Manipulator with Multi-Segment Cables Using Cable Tension Sensor Measurements

**DOI:** 10.3390/s21051623

**Published:** 2021-02-25

**Authors:** Ya’nan Lou, Haoyu Lin, Pengkun Quan, Dongbo Wei, Shichun Di

**Affiliations:** School of Mechatronics Engineering, Harbin Institute of Technology, 150001 Harbin, China; louyn@stu.hit.edu.cn (Y.L.); linhaoyu@hit.edu.cn (H.L.); quanpengkun@hit.edu.cn (P.Q.); weidb@hit.edu.cn (D.W.)

**Keywords:** cable-driven serial manipulators, adaptive robust control, stability analysis, upper bound of uncertainty

## Abstract

The structure of the cable-driven serial manipulator (CDSM) is more complex than that of the cable-driven parallel manipulator (CDPM), resulting in higher model complexity and stronger structural and parametric uncertainties. These drawbacks challenge the stable trajectory-tracking control of a CDSM. To circumvent these drawbacks, this paper proposes a robust adaptive controller for an *n*-degree-of-freedom (DOF) CDSM actuated by *m* cables. First, two high-level controllers are designed to track the joint trajectory under two scenarios, namely known and unknown upper bounds of uncertainties. The controllers include an adaptive feedforward term based on inverse dynamics and a robust control term compensating for the uncertainties. Second, the independence of control gains from the upper bound of uncertainties and the inclusion of the joint viscous friction coefficient into the dynamic parameter vector are realised. Then, a low-level controller is designed for the task of tracking the cable tension trajectory. The system stability is analysed using the Lyapunov method. Finally, the validity and effectiveness of the proposed controllers are verified by experimenting with a three-DOF six-cable CDSM. In addition, a comparative experiment with the classical proportional–integral–derivative (PID) controller is carried out.

## 1. Introduction

Cable-driven serial manipulators (CDSMs) are a series of connected rigid bodies driven by cables instead of actuators which are positioned at each joint [1]. Thus, CDSMs possess low stiffness, low weight, low moving mass and a large reachable workspace. Such robots can be applied in medical rehabilitation [2], assembly in a complex narrow cavity [3,4], automatic charging in electric vehicles [5], nuclear reactors [6], etc. The unidirectional nature of cable actuation (i.e., a cable can only pull and not push) necessitates all the cables be tensed. This paper studies the control of a fully constrained *n*-degree-of-freedom (DOF) CDSM, the actuation cable number m of which is at least n+1, i.e., m≥n+1 [7,8].

Currently, researchers are designing motion controllers for the fully constrained CDSM. One type of controller is based on the kinematic model of the space between the cable and the motor and uses the joint angle as feedback to form a closed-loop control [9,10,11,12]. This type of controller is suitable for scenarios where the robot must move slowly. One type of controller is based on both kinematic and dynamic models of the robot and uses the motor torque obtained from the latter as the feedforward compensation term, which, together with the proportional–integral–derivative (PID) feedback term of the motor position, forms the control input [5,13]. Another type of controller considers not only the positional and trajectory-tracking accuracy of the robot but also the distribution and precise tracking of cable tension. Generally, the distribution of cable tension during the robot’s movement is optimised by taking the minimum cable tension or the minimum joint force and moment as the objective function and the cable tension range and the actual application scenario as the constraints [14,15,16]. However, these control algorithms require knowledge of the robot model based on true kinematic and dynamic parameters. In practice, the kinematic and dynamic models of CDSMs have structural and parametric uncertainties, and the literature does not provide accurate knowledge of the model. These uncertainties limit the tracking performance of the controllers for joint position and cable tension trajectory. A robust adaptive control algorithm could solve this problem.

At present, related studies have proposed robust adaptive controllers for cable-driven parallel manipulators (CDPMs). Khosravi et al. [17] proposed a robust PID controller which requires information about the upper bound of modelling uncertainties. Other studies have designed robust adaptive controllers, assuming that the required knowledge about the bounds of uncertainties is unavailable [18,19,20]. Jabbari and Yoon [19] and Khalilpour et al. [20] proposed a cascade control mechanism for both cable tension and end-effector position. However, the approach in [18] lacks trajectory-tracking control for cable tension and does not provide an adequately quantitative criterion to select the control gains. In [19], the control gains of the outer-loop controller depend on the unavailable practical upper bound of uncertainties. In addition, according to the simulation in that study, the dynamic parameter vector only includes the mass and inertia of the end-effector, while it omits the viscous friction coefficient. This causes its simulation to be inconsistent with the real situation.

However, to the best of the authors’ knowledge, no research has been conducted on robust adaptive algorithms to control the CDSM. In fact, a comparison [21] of the CDSM and the CDPM reveals that the former has a stronger requirement of adaptive algorithms. Obviously, CDSMs are structurally more complex than CDPMs, as they have more controlled links, more cable attachment points and more complicated cable routing paths. Consequently, the CDSM has more structural and parametrical uncertainties. This is the reason there are few accurate models of the CDSM.

Hence, the main objective of this study is to build a theoretical system for stable tracking control of the joint position and cable tension trajectories of the CDSM, considering the uncertainties in the system model, and simultaneously remedying the present drawbacks. The controller design in this paper includes the design of a high-level controller for the tracking control of the joint position trajectory and a low-level controller for the tracking control of the cable tension trajectory. A stability analysis of the entire system with the proposed controller is also conducted. The definition of the whole system and the division of controller hierarchy will be described in detail in Section 3. The innovation of the proposed controllers lies in the following: First, the controllers can work with both known and unknown upper bounds of uncertainties. Second, calculations consider the joint viscous friction coefficient in their dynamic parameter vectors. Third, the high-level controller makes control gains free from the upper bound of uncertainties. Fourth, the low-level controller uses the ball-screw mechanism for tension tracking.

The main contributions of this paper are:

- A robust adaptive controller for the CDSM; a stability analysis of the system as a whole, considering both high-level and low-level controllers; and a performance evaluation of the proposed controller via an experiment with a three-DOF six-cable CDSM, which is the first exploration of the adaptive robust control algorithm for CDSMs so far.
- System modelling for the generalised CDSM, considering the ball-screw transmission mechanism and actuator models is derived. The joint viscous friction coefficient F is included in the dynamic parameter vector π involved in the adaptive law (see Equation (A5)), which is the base of the robust adaptive controller design for CDSMs.
- Compared with the existing research results for CDPMs, the control gains of the high-level controller described in Theorem 2 are independent of the upper bound of uncertainties and other intermediate process parameters. This is also meaningful for the control of CDPMs.
- A performance comparison of two robust adaptive controllers, one with a known upper bound (combination of Theorems 1 and 3) and one without an upper bound of uncertainties (combination of Theorems 2 and 3), is developed.

The remainder of this paper is organised as follows. Section 2 establishes the dynamic model of a generalised n-DOF m-cable CDSM. Section 3 defines the entire system where the control law will be designed and plans the steps of the controller design and stability analysis. Section 4 designs and analyses the stability of high-level and low-level controllers. Section 5 analyses the stability of the entire system. Section 6 validates the designed controller through an experiment with a three-DOF six-cable CDSM. Section 7 concludes the paper with the major findings and future research direction.

## 2. Dynamics Modelling

### 2.1. Dynamics Modelling of the Actuating Cables and Robot Body

**Figure 1:**
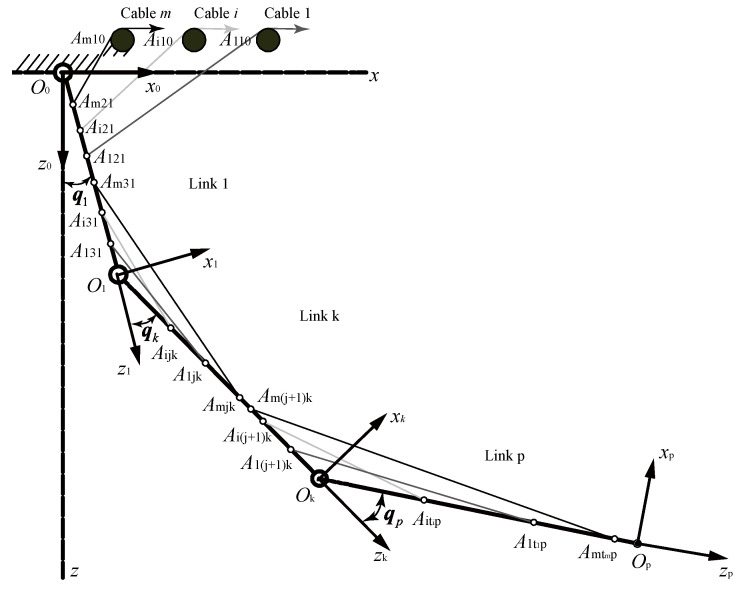
Generalised *n*-degree-of-freedom (DOF) m-cable cable-driven serial manipulator (CDSM).

A p-link CDSM actuated by m cables is illustrated in Figure 1. As shown in the figure, the coordinate frames $O_{0}$, $O_{1}$, $O_{k}$ and $O_{p}$ are attached to the robot base, Link 1, Link k (k=1,⋯,p−1) and Link p, respectively. The symbols $q_{1}$, $q_{k}$ and $q_{p}$ denote the joint space vector of Link 1, Link k and Link p, respectively. The detailed form of $q_{k}$ can be given by $q_{k}={\left[\begin{array}{cccc} q_{k1} & q_{k2} & \ldots & q_{k,n_{k}} \end{array}\right]}^{T}\in {\mathbb{R}}_{n_{k}\times 1}$, where $n_{k}$ denotes the DOF of the corresponding joint (located $O_{k-1}$) of Link k.

For the cable system, the following definitions were applied:

- During the movement of the CDSM, the section where the cable bends is defined as the attachment point.
- The section of the cable between two adjacent attachment points is defined as the cable segment.
- The attachment point $A_{ijk_{j}}$ denotes the j-th ($j=1,\cdots ,t_{i}$) attachment point of Cable i (i=1,⋯,m) on Link $k_{j}$ ($k_{j}=0,1,\cdots ,p$), where $k_{j}$ is the link number where the j-th attachment point is located, $t_{i}$ is the total number of attachment points of Cable i and m is the total number of actuation cables.

The motion equation of the generalised n-DOF m-cable CDSM can be expressed by

(1) $$M\left(q\right)\ddot{q}+C\left(q,\dot{q}\right)\dot{q}+F\dot{q}+g\left(q\right)+\Gamma_{e}=J\left(q\right)\tau_{0},$$

where $q={\left[\begin{array}{cc} q_{1}{}^{T} & \begin{array}{ccc} q_{2}{}^{T} & \ldots & q_{p}{}^{T} \end{array} \end{array}\right]}^{T}\in {\mathbb{R}}_{n\times 1}$ denotes the joint space vector of the CDSM system; $n=\sum_{k=1}^{p}n_{k}$ denotes the total DOF of the CDSM system; and the symbols $M\in {\mathbb{R}}_{n\times n}$, $C\in {\mathbb{R}}_{n\times n}$, $F\in {\mathbb{R}}_{n\times n}$ and $g\in {\mathbb{R}}_{n\times 1}$ denote the inertia matrix, the centrifugal and Coriolis matrices, the diagonal matrix of viscous friction coefficients and the vector of the gravity term, respectively. The symbol $\Gamma_{e}\in {\mathbb{R}}_{n\times 1}$ denotes the joint moments due to the external force and moment $\omega_{e}={\left[\begin{array}{ccc} f_{e1}^{1}{}^{T} & \mu_{e1}^{1}{}^{T} & \begin{array}{ccc} \cdots & f_{ep}^{p}{}^{T} & \mu_{ep}^{p}{}^{T} \end{array} \end{array}\right]}^{T}\in {\mathbb{R}}_{6p\times 1}$ on the entire system, which can be given by

(2) $$\Gamma_{e}=-W^{T}\omega_{e},$$

where the symbol $W\in {\mathbb{R}}_{6p\times n}$ denotes the Jacobian matrix relating external Cartesian wrench $\omega_{e}\in {\mathbb{R}}_{6p\times 1}$ to joint moments $\Gamma_{e}\in {\mathbb{R}}_{n\times 1}$ and the symbols $f_{ek}^{k}\in {\mathbb{R}}_{3\times 1}$ and $\mu_{ek}^{k}\in {\mathbb{R}}_{3\times 1}$ (k=1,⋯,p) denote the external force and moment acting on Link k, respectively. The symbol $J\in {\mathbb{R}}_{n\times m}$ denotes the Jacobian matrix relating input cable tensions $\tau_{0}\in {\mathbb{R}}_{m\times 1}$ to joint moments. It can be given by

(3) $$J=-{\left(VW\right)}^{T}L,$$

where the symbol $V\in {\mathbb{R}}_{n_{s}\times 6p}$ denotes the Jacobian matrix relating cable segment tensions $\tau =\left[\begin{array}{ccc} \tau_{1}{}^{T} & \ldots & \tau_{m}{}^{T} \end{array}\right]\in {\mathbb{R}}_{n_{s}\times 1}$ to the resultant Cartesian wrench $\omega_{T}=\left[\begin{array}{ccc} f_{T1}^{1}{}^{T} & \mu_{T1}^{1}{}^{T} & \begin{array}{ccc} \cdots & f_{Tp}^{p}{}^{T} & \mu_{Tp}^{p}{}^{T} \end{array} \end{array}\right]\in {\mathbb{R}}_{6p\times 1}$ on the CDSM system due to the cable tensions. Here,

(4) $$\omega_{T}=-V^{T}\tau ,$$

where the symbols $f_{Tk}^{k}\in {\mathbb{R}}_{3\times 1}$ and $\mu_{Tk}^{k}\in {\mathbb{R}}_{3\times 1}$ (k=1,⋯,p) denote the resultant force and moment acting on Link k due to the cable tensions, respectively; $\tau_{i}={\left[\begin{array}{ccc} T_{i1} & T_{i2} & \begin{array}{cc} \ldots & T_{i,\;t_{i}-1} \end{array} \end{array}\right]}^{T}\in {\mathbb{R}}_{\left(t_{i}-1\right)\times 1}$ denotes cable tension vector of Cable i; $T_{ij}$ ($j=1,\cdots ,t_{i}-1$) denotes the tension of the j-th segment of Cable i; and $n_{s}=\left(\sum_{i=1}^{m}t_{i}\right)-m$ denotes the total number of cable segments in the CDSM system.

The symbol $L\in {\mathbb{R}}_{n\times m}$ denotes the matrix relating the cable input tensions $\tau_{0}\in {\mathbb{R}}_{m\times 1}$ and the vector of cable segment tensions $\tau \in {\mathbb{R}}_{n_{s}\times 1}$. Here,

(5) $$\tau =L\tau_{0}.$$

The following paragraphs will discuss motion control in free space. Hence, the external force is zero, i.e., $\Gamma_{e}=0$. Based on this assumption, the motion equation described in Equations (1)–(5) has the following properties [22]:

**Property** **1.** *The inertia matrix* M(q) *is symmetric and positive definite.*

**Property** **2.** *The dynamic model can be expressed in linear form with respect to a suitable set of dynamic parameters:*

(6) $$\mu =\Phi \left(q,\dot{q},\ddot{q}\right)\pi =M\left(q\right)\ddot{q}+C\left(q,\dot{q}\right)\dot{q}+F\dot{q}+g\left(q\right)=J\left(q\right)\tau_{0},$$

*where* $\pi \in {\mathbb{R}}_{r\times 1}$ *denotes the vector of constant dynamic parameters and* $\Phi \in {\mathbb{R}}_{n\times r}$ *denotes the regression matrix, which is the function of joint positions, velocities and accelerations.*

**Property** **3.** $\dot{M}\left(q\right)-2C\left(q,\dot{q}\right)$ *is a skew-symmetry matrix, i.e.,*

(7) $$x^{T}\left(\dot{M}\left(q\right)-2C\left(q,\dot{q}\right)\right)x=0,\;\;\forall x\in {\mathbb{R}}_{n\times 1}.$$

### 2.2. Dynamics Modelling of the Transmission Mechanism

**Figure 2:**
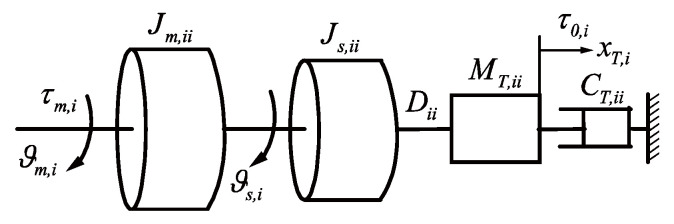
The i-th ball-screw system (i=1,2,⋯,m).

The ball-screw system converts the motor position and output torque into cable lengths and tensions. The model of the i-th ball-screw drive system is drawn in Figure 2. The symbols $\tau_{m,i}$, $\vartheta_{m,i}$, $\vartheta_{s,i}$ and $x_{T,i}$ denote the i-th motor output torque, the i-th motor position, the i-th screw position and the i-th slider position, respectively. The symbols $J_{m,ii}$, $J_{s,ii}$, $M_{T,ii}$ and $C_{T,ii}$ denote the i-th motor–rotor inertia, the moment of inertia of the i-th ball screw, the mass of the i-th slider and the i-th damping coefficient, respectively. The symbol $D_{ii}$ denotes the linear relationship between the i-th motor torque outputs and the i-th cable input tensions.

According to Figure 2, the relationship between the motor torque outputs and the cable input tensions is given by

(8) $$\tau_{m}=D\tau_{0}+\tau_{f},$$

where $D\in {\mathbb{R}}_{m\times m}$ is a diagonal matrix and denotes the linear relationship between the motor torque outputs and cable input tensions, $\tau_{m}\in {\mathbb{R}}_{m\times 1}$ denotes the vector of motor torque outputs and $\tau_{f}\in {\mathbb{R}}_{m\times 1}$ is the torque due to the ball-screw inertia and friction. The i-th diagonal element of D and the i-th element of $\tau_{m}$ are $D_{ii}$ and $\tau_{m,i}$, respectively. The detailed form of $\tau_{f}$ is given by

(9) $$\tau_{f}=J_{m}{\ddot{\vartheta }}_{m}+J_{s}{\ddot{\vartheta }}_{s}-D\left(M_{T}{\ddot{x}}_{T}+C_{T}{\dot{x}}_{T}\right),$$

where $\vartheta_{m}$, $\vartheta_{s}$ and $x_{T}$ denote the motor position, screw position and slider position, respectively. $J_{m}$, $J_{s}$, $M_{T}$ and $C_{T}$ denote the motor–rotor inertia, the moment of inertia of the ball screw, the mass of the slider and the damping coefficient, respectively, which are all diagonal matrices. The i-th element of $\vartheta_{m}$, $\vartheta_{s}$, $x_{T}$ and the i-th diagonal element of $J_{m}$, $J_{s}$, $M_{T}$, $C_{T}$ are $\vartheta_{m,i}$, $\vartheta_{s,i}$, $x_{T,i}$, $J_{m,ii}$, $J_{s,ii}$, $M_{T,ii}$ and $C_{T,ii}$, respectively.

## 3. System Overview

The system model described by Equations (1) and (8) can be regarded as a cascaded structure. Two controllers, i.e., high-level and low-level controllers, were designed to control this system. The high-level controller outs the desired input cable tensions $\tau_{0,d}$, which can track the desired joint trajectory $q_{d}$. The outputs of the high-level controller are regarded as inputs of the low-level controller. The low-level controller outs the motor commands $\tau_{m}$ (desired motor torque outputs) which ensure that the cable tension follows $\tau_{0,d}$ and are the motors inputs. By introducing the virtual control vector μ, the CDSM system model can be described by two first-order differential matrix equations:

(10) $$\begin{array}{l} {\dot{e}}_{q}=\underset{f_{e_{q}}\left(e_{q},\mu ,q,\dot{q},\ddot{q}\right)}{\underbrace{S_{1}e_{q}+S_{2}M^{-1}\left(\mu_{r}-\mu \right)}}+\underset{f_{\Delta }\left(\mu ,{\tilde{\tau }}_{0},\tau_{0,d}\right)}{\underbrace{S_{2}M^{-1}\left(J{\tilde{\tau }}_{0}-J\tau_{0,d}+\mu \right)}}\\ {\dot{e}}_{\tau_{0}}=\underset{f_{e_{\tau_{0}}}\left(e_{\tau_{0}},{\ddot{\tau }}_{m},{\ddot{\tau }}_{0,d},{\ddot{\tau }}_{f}\right)}{\underbrace{S_{1}e_{\tau_{0}}+S_{2}\left({\ddot{\tau }}_{0,d}-D^{-1}\left({\ddot{\tau }}_{m}-{\ddot{\tau }}_{f}\right)\right)}} \end{array},$$

where $\mu_{r}$ is given by $M\left(q\right){\ddot{q}}_{d}+C\left(q,\dot{q}\right)\dot{q}+F\dot{q}+g\left(q\right)$, and

(11) $$e_{q}={\left[\begin{array}{cc} {\tilde{q}}^{T} & {\dot{\tilde{q}}}^{T} \end{array}\right]}^{T},\;e_{\tau_{0}}={\left[\begin{array}{cc} {\tilde{\tau }}_{0}^{T} & {\dot{\tilde{\tau }}}_{0}^{T} \end{array}\right]}^{T}$$

denote the tracking error and are taken as the system state; $\tilde{q}=q_{d}-q$ and ${\tilde{\tau }}_{0}=\tau_{0,d}-\tau_{0}$ denote the joint position error and the cable input tensions error, respectively; and the symbols $S_{1}\in {\mathbb{R}}_{2n\times 2n}$ and $S_{2}\in {\mathbb{R}}_{2n\times n}$ denote block matrices and are given by

(12) $$S_{1}=\left[\begin{array}{cc} O_{n\times n} & I_{n\times n}\\ O_{n\times n} & O_{n\times n} \end{array}\right],\;S_{2}=\left[\begin{array}{c} O_{n\times n}\\ I_{n\times n} \end{array}\right],$$

respectively, where $I_{n\times n}$ denotes a n×n identity matrix.

In the following sections, on the assumption that the coupling term $f_{\Delta }$ can be ignored, the controller design and stability of the two differential equation systems in Equation (10) are demonstrated. Then, under the assumption that the coupling term $f_{\Delta }$ cannot be ignored, the stability of the entire system represented by Equation (10) is proved under the action of the above controllers.

For the sake of clarity, the key mathematical symbols used in the controller design and their physical meanings are shown in Table 1.

## 4. Adaptive Robust Control

### 4.1. Adaptive Robust Control of Cable Tensions

The research object in this subsection is a high-level subsystem which ignores the coupling term $f_{\Delta }$, i.e., ${\dot{e}}_{q}=f_{e_{q}}\left(e_{q},\mu ,q,\dot{q},\ddot{q}\right)$, which can be rewritten as

(13) $${\dot{e}}_{q}=S_{1}e_{q}+\left(-S_{2}M^{-1}\right)\mu +\left(-S_{2}M^{-1}\right)\left(-\mu_{r}\right),$$

and further expanded to

(14) $$M\left(q\right)\ddot{q}+C\left(q,\dot{q}\right)\dot{q}+F\dot{q}+g\left(q\right)=\mu .$$

The purposes of the controller for this subsystem are to design the control input μ and obtain the positive input cable tension $\tau_{0}$ to ensure that the joint-position-tracking error $e_{q}$ asymptotically converges to zero or is uniformly ultimately bounded (UUB).

The proposed control law is given by

(15) $$\begin{array}{ll} \mu & =\hat{M}\left(q\right){\ddot{q}}_{r}+\hat{C}\left(q,\dot{q}\right){\dot{q}}_{r}+\hat{F}{\dot{q}}_{r}+\hat{g}\left(q\right)+K_{D}\sigma_{q}+\omega \\ & =\Phi \left(q,\dot{q},{\dot{q}}_{r},{\ddot{q}}_{r}\right)\hat{\pi }+K_{D}\sigma_{q}+\omega =\hat{J}\left(q\right)\tau_{0} \end{array},$$

where ${\dot{q}}_{r}$, ${\ddot{q}}_{r}$ and $\sigma_{q}$ are given by

(16) $${\dot{q}}_{r}={\dot{q}}_{d}+\Lambda \tilde{q},\;\;{\ddot{q}}_{r}={\ddot{q}}_{d}+\Lambda \dot{\tilde{q}},\;\sigma_{q}=\dot{\tilde{q}}+\Lambda \tilde{q}={\dot{q}}_{r}-\dot{q}.$$

with a diagonal positive definite matrix Λ. $K_{D}$ denotes the diagonal control gain matrix, ω denotes the robust term and $\hat{M}$, $\hat{C}$, $\hat{F}$, $\hat{g}$ and $\hat{\pi }$ denote the estimates of M, C, F, g and π, respectively.

The general solution of $\tau_{0}$ in Equation (15) can be given by

(17) $$\tau_{0}={\hat{J}}^{†}\mu +N\lambda ,$$

where $\lambda \in {\mathbb{R}}_{n\times 1}$ is an underdetermined vector, $\hat{J}$ denotes the estimate of J, ${\hat{J}}^{†}$ denotes the peso-inverse of $\hat{J}$ and is given by ${\hat{J}}^{†}={\hat{J}}^{T}{\left(\hat{J}{\hat{J}}^{T}\right)}^{-1}\;\mathrm{and}\;N$ denotes the kernel of $\hat{J}$, assuming matrix $\hat{J}$ is full rank.

There are two different ways to design the estimate $\hat{\pi }$ and the robust term ω. The first way is given as the robust term described by

(18) $$\omega =\frac{\rho }{\left\|\sigma_{q}\right\|}\sigma_{q},$$

and the parameter adaptive law described by

(19) $$\dot{\hat{\pi }}=K_{\pi }^{-1}\Phi^{T}\left(q,\dot{q},{\dot{q}}_{r},{\ddot{q}}_{r}\right)\sigma_{q},$$

where $\rho \in {\mathbb{R}}^{+}$ denotes the adjustable gain and $K_{\pi }$ denotes the diagonal control gain matrix.

Another way is by describing the robust term as

(20) $$\omega =\frac{{\hat{\varsigma }}^{2}\sigma_{q}}{\|\sigma_{q}\|\hat{\varsigma }+\epsilon_{2}},$$

and the parameter adaptive law as

(21) $$\dot{\hat{\pi }}=Proj\left(K_{\pi }{}^{-1}\Phi^{T}\left(q,\dot{q},{\dot{q}}_{r},{\ddot{q}}_{r}\right)\sigma_{q}+\beta_{0}K_{\pi }{}^{-1}\hat{\pi }\right),$$

(22) $$\dot{\hat{\varsigma }}=Proj\left(\frac{1}{\lambda }\|\sigma_{q}\|-\beta \frac{1}{\lambda }\hat{\varsigma }\right),$$

where $\varsigma =\xi_{1}+\xi_{2}\in {\mathbb{R}}^{+}$ denotes the upper bound of uncertainties, $\xi_{1}\in {\mathbb{R}}^{+}$ and $\xi_{2}\in {\mathbb{R}}^{+}$ will be expressed in the subsequent paragraphs and $\beta_{0}\in {\mathbb{R}}^{+}$, $\beta \in {\mathbb{R}}^{+}$, $\lambda \in {\mathbb{R}}^{+}$ and $\epsilon_{2}\in {\mathbb{R}}^{+}$ are the adjustable parameters. The operation Proj(·) is a continuous projection function, detailed information about which can be found in [23].

Therefore, the following two theorems on stability analysis for joint position trajectory tracking are formed:

**Theorem** **1.** *The trajectories of the CDSM are described by the model in Equation (14) under the control law in Equations (15) and (17), the robust control law described by Equation (18), the parameter adaptive law described by Equations (19) and (16) and the following sufficient condition*

(23) $$\rho \geq \xi_{1}+\xi_{2}$$

*globally asymptotically converges to* $\sigma_{q}=0$ *and* $\tilde{q}=0$ *, which implies convergence of* $\tilde{q}$ *and* $\dot{\tilde{q}}$ *to zero and the boundedness of* $\hat{\pi }$ *.*

**Theorem** **2.** *The tracking error trajectories of the CDSM described by the model in Equation (14) under the control law in Equations (15) and (17), the robust control law described by Equation 21, the parameter adaptive law described by Equations (21), (16) and (22) and the following sufficient condition*

(24) $$\begin{array}{l} \alpha_{ii}>\frac{1}{2}\\ \begin{array}{l} K_{D,ii}>0,\;\;\;\;\;\;\;\;if\;\frac{1}{2}<\alpha_{ii}\leq 1\\ K_{D,ii}<\frac{F_{ii}}{\alpha_{ii}-1},\;\;if\;\alpha_{ii}>1\;\;\;\;\;\;\;\; \end{array} \end{array}$$

*is UUB, where* $F_{ii}$ *,* $\alpha_{ii}$ *and* $K_{D,ii}$ *denote the* i *-th (* i=1,⋯,n *) diagonal element of* F *,* Λ *and* $K_{D}$ *, respectively.*

Obviously, when $1/2<\alpha_{ii}\leq 1$, the choice of $K_{D,ii}$ is independent of the upper bound of uncertainties and other intermediate process parameters. Notice that when $\alpha_{ii}>1$, the choice of $K_{D,ii}$ depends on the viscous friction coefficient $F_{ii}$. However, the true value of $F_{ii}$ is unavailable. If the lower bound value of $F_{ii}$ is available, i.e., there is a $F_{ii,l}$ such that $F_{ii}\geq F_{ii,l}$ holds, then the value range of $K_{D,ii}$ can be redefined as $K_{D,ii}\leq F_{ii,l}/\left(\alpha_{ii}-1\right)$.

**Remark** **1.** *For the robust control law, Equation (18) in Theorem 1 requires prior knowledge of the upper bound of uncertainties* ς *, whereas Equation (20) of Theorem 2 does not. Compared with the control gain in [19], which depends on the upper bound of uncertainties of the system, the control gains described by Equation (24) are independent of this upper bound. Hence, the high-level controller described in Theorem 2 eliminates the requirement of knowing the upper bound of uncertainties. With the premise of zero loss of robustness of the adaptive law, this could not be achieved in [19].*

**Proof** **of** **Theorem** **1.** Substituting Equation (15) into the dynamics model of the actuating cables and robot body described by Equation (6) gives

(25) $$M\left(q\right)\ddot{q}+C\left(q,\dot{q}\right)\dot{q}+F\dot{q}+g\left(q\right)=\hat{M}\left(q\right){\ddot{q}}_{r}+\hat{C}\left(q,\dot{q}\right){\dot{q}}_{r}+\hat{F}{\dot{q}}_{r}+\hat{g}\left(q\right)+K_{D}\sigma_{q}+\omega .$$

Subtracting $M\left(q\right){\ddot{q}}_{r}+C\left(q,\dot{q}\right){\dot{q}}_{r}+F{\dot{q}}_{r}+g\left(q\right)$ in the left and right parts of Equation (25) gives

(26) $$M\left(q\right){\dot{\sigma }}_{q}+C\left(q,\dot{q}\right)\sigma_{q}+F\sigma_{q}+K_{D}\sigma_{q}+\omega =-\tilde{M}\left(q\right){\ddot{q}}_{r}-\tilde{C}\left(q,\dot{q}\right){\dot{q}}_{r}-\tilde{F}{\dot{q}}_{r}-\tilde{g}\left(q\right)=-\Phi \left(q,\dot{q},{\dot{q}}_{r},{\ddot{q}}_{r}\right)\tilde{\pi },$$

where ${\dot{q}}_{r}-\dot{q}=\sigma_{q}$ and ${\ddot{q}}_{r}-\ddot{q}={\dot{\sigma }}_{q}$ are exploited and $\tilde{M}$, $\tilde{C}$, $\tilde{F}$ and $\tilde{g}$ denote the modelling error and can be given by $\tilde{M}=\hat{M}-M,\;\tilde{C}=\hat{C}-C,\;\tilde{F}=\hat{F}-F\;\mathrm{and}\;\tilde{g}=\hat{g}-g$, respectively.

The property of linearity shown in Property 2 is exploited. Hence, the vector of parameter error is $\tilde{\pi }=\hat{\pi }-\pi$.

Consider the Lyapunov function candidate, which is given by

(27) $$V\left(\sigma_{q},\;\tilde{q},\;\tilde{\pi }\right)=\frac{1}{2}\sigma_{q}{}^{T}M\left(q\right)\sigma_{q}+{\tilde{q}}^{T}\Lambda K_{D}\tilde{q}+\frac{1}{2}{\tilde{\pi }}^{T}K_{\pi }\tilde{\pi }>0,\;\;\forall \sigma_{q},\tilde{q},\;\tilde{\pi }\neq 0.$$

The time derivative of V can be obtained as

(28) $$\begin{array}{ll} \dot{V} & =\sigma_{q}{}^{T}M\left(q\right){\dot{\sigma }}_{q}+\frac{1}{2}\sigma_{q}{}^{T}\dot{M}\left(q\right)\sigma_{q}+2{\tilde{q}}^{T}\Lambda K_{D}\dot{q}+{\tilde{\pi }}^{T}K_{\pi }\dot{\tilde{\pi }}\\ & =\sigma_{q}{}^{T}\left[M\left(q\right){\dot{\sigma }}_{q}+\frac{1}{2}\left(\dot{M}\left(q\right)-2C\left(q,\dot{q}\right)\right)\sigma_{q}+C\left(q,\dot{q}\right)\sigma_{q}\right]+2{\tilde{q}}^{T}\Lambda K_{D}\dot{\tilde{q}}+{\tilde{\pi }}^{T}K_{\pi }\dot{\tilde{\pi }} \end{array}.$$

We modify Equation (28) as follows: apply Property 3, expand ${\dot{\sigma }}_{q}$ as $\left({\ddot{q}}_{d}-\ddot{q}\right)+\Lambda \dot{\tilde{q}}$ and then substitute the generated $-M\left(q\right)\ddot{q}$ with $C\left(q,\dot{q}\right)\dot{q}+F\dot{q}+g\left(q\right)-J\left(q\right)\tau_{0}$ according to Equation (6) to get

(29) $$\dot{V}=\sigma_{q}{}^{T}\left[M\left(q\right){\ddot{q}}_{r}+C\left(q,\dot{q}\right){\dot{q}}_{r}+F\dot{q}+g\left(q\right)-J\left(q\right)\tau_{0}\right]+2{\tilde{q}}^{T}\Lambda K_{D}\dot{\tilde{q}}+{\tilde{\pi }}^{T}K_{\pi }\dot{\tilde{\pi }},$$

where ${\ddot{q}}_{r}={\ddot{q}}_{d}+\Lambda \dot{\tilde{q}}$ and ${\dot{q}}_{r}=\dot{q}+\sigma_{q}$ are exploited.

Substituting Equation (17), and then substituting Equation (15) into Equation (29), gives

(30) $$\begin{array}{ll} \dot{V} & =\sigma_{q}{}^{T}\left[M{\ddot{q}}_{r}+C{\dot{q}}_{r}+F\dot{q}+g-J\left({\hat{J}}^{†}\mu +N\lambda \right)\right]+2{\tilde{q}}^{T}\Lambda K_{D}\dot{\tilde{q}}+{\tilde{\pi }}^{T}K_{\pi }\dot{\tilde{\pi }}\\ & =\sigma_{q}{}^{T}\left[M{\ddot{q}}_{r}+C{\dot{q}}_{r}+F\dot{q}+g-J{\hat{J}}^{†}\mu +\mu -\mu -JN\lambda +\hat{J}N\lambda \right]+2{\tilde{q}}^{T}\Lambda K_{D}\dot{\tilde{q}}+{\tilde{\pi }}^{T}K_{\pi }\dot{\tilde{\pi }}\\ & =\sigma_{q}{}^{T}\left[-\tilde{M}{\ddot{q}}_{r}-\tilde{C}{\dot{q}}_{r}-\tilde{g}-\hat{F}{\dot{q}}_{r}+F\dot{q}-K_{D}\sigma_{q}-\omega +\eta \right]+2{\tilde{q}}^{T}\Lambda K_{D}\dot{\tilde{q}}+{\tilde{\pi }}^{T}K_{\pi }\dot{\tilde{\pi }} \end{array},$$

where $\hat{J}N\lambda =0$ is exploited and

(31) $$\eta =\left(I-J{\hat{J}}^{†}\right)\mu +\left(\hat{J}-J\right)N\lambda$$

is a nonlinear function of $\tilde{q}$ and $\dot{\tilde{q}}$. Actually, η involves the real and estimated values of the coordinates of the cable attachment points and the link parameters. Therefore, η can indicate the uncertainty to which the controller must robust.

Substituting Equation (26) into Equation (30) and then applying Equation (16) in the term $-\sigma_{q}{}^{T}K_{D}\sigma_{q}$ gives $\dot{V}$ along the trajectories of system Equation (26), which is given by

(32) $$\begin{array}{ll} \dot{V} & =\sigma_{q}{}^{T}\left[-\Phi \left(q,\dot{q},{\dot{q}}_{r},{\ddot{q}}_{r}\right)\tilde{\pi }+\tilde{F}{\dot{q}}_{r}-\hat{F}{\dot{q}}_{r}+F\dot{q}-K_{D}\sigma_{q}-\omega +\eta \right]+2{\tilde{q}}^{T}\Lambda K_{D}\dot{\tilde{q}}+{\tilde{\pi }}^{T}K_{\pi }\dot{\tilde{\pi }}\\ & =\sigma_{q}{}^{T}\left[-\Phi \left(q,\dot{q},{\dot{q}}_{r},{\ddot{q}}_{r}\right)\tilde{\pi }-F\sigma_{q}-K_{D}\sigma_{q}-\omega +\eta \right]+2{\tilde{q}}^{T}\Lambda K_{D}\dot{\tilde{q}}+{\tilde{\pi }}^{T}K_{\pi }\dot{\tilde{\pi }}\\ & ={\tilde{\pi }}^{T}\left(K_{\pi }\dot{\tilde{\pi }}-\Phi^{T}\left(q,\dot{q},{\dot{q}}_{r},{\ddot{q}}_{r}\right)\sigma_{q}\right)-\sigma_{q}{}^{T}K_{D}\sigma_{q}+2{\tilde{q}}^{T}\Lambda K_{D}\dot{\tilde{q}}+\sigma_{q}{}^{T}\left(\eta -\omega \right)-\sigma_{q}{}^{T}F\sigma_{q}\\ & ={\tilde{\pi }}^{T}\left(K_{\pi }\dot{\tilde{\pi }}-\Phi^{T}\left(q,\dot{q},{\dot{q}}_{r},{\ddot{q}}_{r}\right)\sigma_{q}\right)-{\dot{\tilde{q}}}^{T}K_{D}\dot{\tilde{q}}-{\tilde{q}}^{T}\Lambda K_{D}\Lambda \tilde{q}+\sigma_{q}{}^{T}\left(\eta -\omega \right)-\sigma_{q}{}^{T}F\sigma_{q} \end{array}.$$

If the estimate of the parameter vector is updated as in the adaptive law described by Equation (19), the representation of Equation (32) becomes

(33) $$\dot{V}=\sigma_{q}{}^{T}\left(\eta -\omega \right)-\sigma_{q}{}^{T}F\sigma_{q}-{\dot{\tilde{q}}}^{T}K_{D}\dot{\tilde{q}}-{\tilde{q}}^{T}\Lambda K_{D}\Lambda \tilde{q},$$

where $\dot{\tilde{\pi }}=\dot{\hat{\pi }}$ is exploited.

Consider that, for $\forall \;\sigma_{q},\tilde{q},\;\dot{\tilde{q}}\neq 0$, there exists $-\sigma_{q}{}^{T}F\sigma_{q}-{\dot{\tilde{q}}}^{T}K_{D}\dot{\tilde{q}}-{\tilde{q}}^{T}\Lambda K_{D}\Lambda \tilde{q}<0$.

However, the signum of $\sigma_{q}{}^{T}\left(\eta -\omega \right)$ is uncertain. This may imply that the negative value of Equation (33) cannot be guaranteed. The term ω should be chosen to guarantee robustness to the effects of the uncertainty η described by Equation (31). Hence, the selection of the robust contribution ω, which renders $\sigma_{q}{}^{T}\left(\eta -\omega \right)$ less than or equal to zero, is the following work of this proof. We assume that even though the uncertainty η is unknown, the range of its variation can be estimated. Based on this assumption, adopting the control law described by Equation (18) gives

(34) $$\sigma_{q}{}^{T}\left(\eta -\omega \right)=\sigma_{q}{}^{T}\eta -\frac{\rho }{\left\|\sigma_{q}\right\|}\sigma_{q}{}^{T}\sigma_{q}=\sigma_{q}{}^{T}\eta -\rho \|\sigma_{q}\|\leq \|\sigma_{q}\|\|\eta \|-\rho \|\sigma_{q}\|=\|\sigma_{q}\|\left(\|\eta \|-\rho \right).$$

Then, if ρ is chosen so that

(35) $$\rho \geq \|\eta \|,\;\;\;\;\;\;\;\;\;\;\;\;\;\;\;\forall q,\dot{q},{\dot{q}}_{d},{\ddot{q}}_{d}$$

the control law Equation (15) ensures that $\dot{V}$ is less than zero along all error system trajectories. To satisfy Equation (35), the uncertainty is

(36) $$\|\eta \|\leq \left\|(I-J{\hat{J}}^{†}\right)\mu \|+\left\|(\hat{J}-J\right)N\lambda \|\leq \xi_{1}+\xi_{2},$$

where $\left\|(I-J{\hat{J}}^{†}\right)\mu \|\leq \xi_{1},\left\|(\hat{J}-J\right)N\lambda \|\leq \xi_{2}$.

It is easy to see that by satisfying the condition of Equation (23), ρ can give

(37) $$\dot{V}=\sigma_{q}{}^{T}\left(\eta -\omega \right)-\sigma_{q}{}^{T}F\sigma_{q}-{\dot{\tilde{q}}}^{T}K_{D}\dot{\tilde{q}}-{\tilde{q}}^{T}\Lambda K_{D}\Lambda \tilde{q}<0,\;\;\;\;\forall \;\sigma_{q},\tilde{q},\;\dot{\tilde{q}}\neq 0.$$

Hence, the negative definite of the time derivative of V ensures global asymptotic stability of the equilibrium $\sigma_{q}=0$ and $\tilde{q}=0$. □

**Proof** **of** **Theorem** **2.** Consider the Lyapunov function candidate

(38) $$V\left(\sigma_{q},\tilde{q},\;\tilde{\pi },\tilde{\varsigma }\right)=\frac{1}{2}\sigma_{q}{}^{T}M\left(q\right)\sigma_{q}+{\tilde{q}}^{T}\Lambda K_{D}\tilde{q}+\frac{1}{2}{\tilde{\pi }}^{T}K_{\pi }\tilde{\pi }+\frac{1}{2}\lambda {\tilde{\varsigma }}^{2}>0,\;\;\forall \sigma_{q},\tilde{q},\;\tilde{\pi },\neq 0,\;\tilde{\varsigma }\neq 0,$$

where $\tilde{\varsigma }=\hat{\varsigma }-\varsigma$ denotes the error of the upper bound of uncertainties.

This function can be bounded as

(39) $$\gamma_{1}\left\|(e)\right\|\leq V\leq \gamma_{2}\left\|(e)\right\|,$$

where $\gamma_{1}\left\|(e)\right\|=\eta_{l}\|(e){\|}^{2}$ and $\gamma_{2}\left\|(e)\right\|=\eta^{u}\|(e){\|}^{2}$ with $e={\left(\sigma_{q}{}^{T},\;{\tilde{q}}^{T},{\tilde{\pi }}^{T},{\tilde{\varsigma }}^{T}\right)}^{T}$, $\eta_{l}=min\left(\frac{1}{2}\lambda_{min}\left(M\right),\lambda_{min}\left(\Lambda K_{D}\right),\frac{1}{2}\lambda_{min}\left(K_{\pi }\right),\frac{1}{2}\lambda \right)$ and $\eta^{u}=max\left(\frac{1}{2}\lambda_{max}\left(M\right),\lambda_{max}\left(\Lambda K_{D}\right),\frac{1}{2}\lambda_{max}\left(K_{\pi }\right),\frac{1}{2}\lambda \right)$, with $\lambda_{max}\left(\cdot \right)$ and $\lambda_{min}\left(\cdot \right)$ denoting, respectively, the maximum and minimum eigenvalues of a matrix.

Based on Equation (32), the time derivative of V is given by

(40) $$\dot{V}={\tilde{\pi }}^{T}\left(K_{\pi }\dot{\tilde{\pi }}-\Phi^{T}\left(q,\dot{q},{\dot{q}}_{r},{\ddot{q}}_{r}\right)\sigma_{q}\right)+\sigma_{q}{}^{T}\left(\eta -\omega \right)-\sigma_{q}{}^{T}F\sigma_{q}-{\dot{\tilde{q}}}^{T}K_{D}\dot{\tilde{q}}-{\tilde{q}}^{T}\Lambda K_{D}\Lambda \tilde{q}+\lambda \tilde{\varsigma }\dot{\hat{\varsigma }},$$

where $\dot{\hat{\varsigma }}=\dot{\tilde{\varsigma }}$ is exploited.

According to Equation (21), the following inequality can be given as

(41) $$-\sigma_{q}{}^{T}\omega =-\frac{\|\sigma_{q}{\|}^{2}{\hat{\varsigma }}^{2}}{\|\sigma_{q}\|\hat{\varsigma }+\epsilon_{2}}\leq -\|\sigma_{q}\|\hat{\varsigma }+\epsilon_{2}.$$

Substituting Equation (41) into Equation (40), $\dot{V}$ can be bounded as

(42) $$\begin{array}{ll} \dot{V} & \leq {\tilde{\pi }}^{T}\left(K_{\pi }\dot{\tilde{\pi }}-\Phi^{T}\left(q,\dot{q},{\dot{q}}_{r},{\ddot{q}}_{r}\right)\sigma_{q}\right)+\sigma_{q}{}^{T}\eta -\sigma_{q}{}^{T}F\sigma_{q}-{\dot{\tilde{q}}}^{T}K_{D}\dot{\tilde{q}}-{\tilde{q}}^{T}\Lambda K_{D}\Lambda \tilde{q}+\lambda \tilde{\varsigma }\dot{\hat{\varsigma }}-\|\sigma_{q}\|\hat{\varsigma }+\epsilon_{2}\\ & \leq {\tilde{\pi }}^{T}\left(K_{\pi }\dot{\tilde{\pi }}-\Phi^{T}\left(q,\dot{q},{\dot{q}}_{r},{\ddot{q}}_{r}\right)\sigma_{q}\right)+\|\sigma_{q}\|\|\eta \|-\sigma_{q}{}^{T}F\sigma_{q}-{\dot{\tilde{q}}}^{T}K_{D}\dot{\tilde{q}}-{\tilde{q}}^{T}\Lambda K_{D}\Lambda \tilde{q}+\lambda \tilde{\varsigma }\dot{\hat{\varsigma }}-\|\sigma_{q}\|\hat{\varsigma }+\epsilon_{2}\\ & \leq {\tilde{\pi }}^{T}\left(K_{\pi }\dot{\tilde{\pi }}-\Phi^{T}\left(q,\dot{q},{\dot{q}}_{r},{\ddot{q}}_{r}\right)\sigma_{q}\right)-\sigma_{q}{}^{T}F\sigma_{q}-{\dot{\tilde{q}}}^{T}K_{D}\dot{\tilde{q}}-{\tilde{q}}^{T}\Lambda K_{D}\Lambda \tilde{q}+\lambda \tilde{\varsigma }\dot{\hat{\varsigma }}+\|\sigma_{q}\|\varsigma -\|\sigma_{q}\|\hat{\varsigma }+\epsilon_{2}\\ & ={\tilde{\pi }}^{T}\left(K_{\pi }\dot{\tilde{\pi }}-\Phi^{T}\left(q,\dot{q},{\dot{q}}_{r},{\ddot{q}}_{r}\right)\sigma_{q}\right)-\sigma_{q}{}^{T}F\sigma_{q}-{\dot{\tilde{q}}}^{T}K_{D}\dot{\tilde{q}}-{\tilde{q}}^{T}\Lambda K_{D}\Lambda \tilde{q}+\tilde{\varsigma }\left(\lambda \dot{\hat{\varsigma }}-\|\sigma_{q}\|\right)+\epsilon_{2} \end{array},$$

where Equation (36) is exploited.

Substituting the parameter adaptive law described by Equations (21) and (22) into Equation (42), and applying the following inequalities $\beta_{0}{\tilde{\pi }}^{T}\hat{\pi }\leq -\frac{\beta_{0}}{2}\|\tilde{\pi }{\|}^{2}+\frac{\beta_{0}}{2}\|\pi {\|}^{2}$, $\beta \tilde{\varsigma }\hat{\varsigma }\leq -\frac{\beta }{2}{\tilde{\varsigma }}^{2}+\frac{\beta }{2}\varsigma^{2}$ gives the bounded $\dot{V}$ described by

(43) $$\dot{V}\leq -\sigma_{q}{}^{T}F\sigma_{q}-{\dot{\tilde{q}}}^{T}K_{D}\dot{\tilde{q}}-{\tilde{q}}^{T}\Lambda K_{D}\Lambda \tilde{q}-\frac{\beta_{0}}{2}\|\tilde{\pi }{\|}^{2}+\frac{\beta_{0}}{2}\|\pi {\|}^{2}-\frac{\beta }{2}{\tilde{\varsigma }}^{2}+\frac{\beta }{2}\varsigma^{2}+\epsilon_{2}.$$

It is reasonable to assume that $\|\pi \|\leq \pi_{M}$ on the dynamic parameter vector, where $\pi_{M}$ denotes the upper bound of the norm of π. Hence, further, Equation (43) can be written as

(44) $$\dot{V}\leq -\delta^{T}Q\delta -\frac{\beta_{0}}{2}\|\tilde{\pi }{\|}^{2}-\frac{\beta }{2}{\tilde{\varsigma }}^{2}+\chi ,$$

where $\chi =\frac{\beta_{0}}{2}\pi_{M}{}^{2}+\frac{\beta }{2}\varsigma^{2}+\epsilon_{2}\geq \frac{\beta_{0}}{2}\|\pi {\|}^{2}+\frac{\beta }{2}\varsigma^{2}+\epsilon_{2}$ is exploited, δ is given by $\delta ={\left[\begin{array}{cc} \sigma_{q}{}^{T} & {\tilde{q}}^{T} \end{array}\right]}^{T}$ and ***Q*** is given by

(45) $$Q=\left[\begin{array}{cc} F+\left(I-\Lambda^{T}\right)K_{D} & O\\ O & \Lambda K_{D}\Lambda +\Lambda^{T}K_{D}\left(\Lambda -I\right) \end{array}\right].$$

To ensure that Q is positive definite, the following conditions must be satisfied:

(46) $$\begin{array}{c} F_{ii}+\left(1-\alpha_{ii}\right)K_{D,ii}>0\\ K_{D,ii}\alpha_{ii}\left(2\alpha_{ii}-1\right)>0 \end{array},$$

where $F_{ii},\;\alpha_{ii}$ and $K_{D,ii}$ denote the i-th (i=1,⋯,n) diagonal elements of F, Λ and $K_{D}$, respectively. Further, the conditions described by Equation (24) can be obtained.

Based on these conditions, $\dot{V}$ in Equation (44) can be bounded as

(47) $$\dot{V}\leq -\gamma_{3}\left(\|e\|\right)+\chi ,$$

where $\gamma_{3}\left(\|e\|\right)=\beta_{1}\|e{\|}^{2}$ with $\beta_{1}=min\left(\lambda_{min}\left(Q\right),\frac{\beta_{0}}{2},\frac{\beta }{2}\right)$, and $\|e{\|}^{2}=\|\sigma q{\|}^{2}+\|\tilde{q}{\|}^{2}+\|\tilde{\pi }{\|}^{2}+{\tilde{\varsigma }}^{2}=\|\delta {\|}^{2}+\|\tilde{\pi }{\|}^{2}+{\tilde{\varsigma }}^{2}$ is exploited.

Now, given Equations (47) and (39), provided the condition in Equation (24) is satisfied, e is UUB, according to the Lyapunov theorem extension [24,25], in the sense that $\|\tilde{q}\|\leq \|e\|<r,\;\;\;\;\;\forall t\geq T\left(r,\|e\left(0\right)\|\right)$, where $r\in {\mathbb{R}}^{+}$ denotes the radius of a ball containing the joint-position-tracking errors, which can be selected as

(48) $$r>\left(\gamma_{1}{}^{-1}\circ \gamma_{2}\right)\left(\gamma_{3}{}^{-1}\left(\chi \right)\right),$$

and $T\in {\mathbb{R}}^{+}$ is a constant which states the time to reach the ball, which is given by

(49) $$T=\{\begin{array}{ll} 0, & \|e\left(0\right)\|\leq \left(\gamma_{2}{}^{-1}\circ \gamma_{1}\right)\left(r\right)\\ \frac{\gamma_{2}(\left\|e\left(0\right)\right\|)-\gamma_{1}\left(\gamma_{2}{}^{-1}\circ \gamma_{1}\left(r\right)\right)}{\gamma_{3}\left(\gamma_{2}{}^{-1}\circ \gamma_{1}\left(r\right)\right)-\chi }, & \|e\left(0\right)\|>\left(\gamma_{2}{}^{-1}\circ \gamma_{1}\right)\left(r\right) \end{array},$$

where $\gamma_{1}{}^{-1}\circ \gamma_{2}\left(\|e\|\right)=\sqrt{\frac{\eta^{u}}{\eta_{l}}}\|e\|$, $\gamma_{3}{}^{-1}\left(\chi \right)=\sqrt{\frac{\chi }{\beta_{1}}}$ and $\gamma_{2}{}^{-1}\circ \gamma_{1}\left(\|e\|\right)=\sqrt{\frac{\eta_{l}}{\eta^{u}}}\|e\|$. Substituting them into Equations (48) and (49) gives

(50) $$r>\sqrt{\frac{\eta^{u}}{\eta_{l}\beta_{1}}}\sqrt{\chi }.$$

Taken r to be $\sqrt{\beta_{2}\chi }$, and then substituting it into Equation (49) gives

(51) $$T=\{\begin{array}{c} 0,\;\;\;\;\;\;\;\;\;\;\;\;\;\;\;\;\;\;\;\;\;\;\;\;\;\;\;\|e\left(0\right)\|\leq \sqrt{\beta_{2}\frac{\eta_{l}}{\eta^{u}}\chi }\\ \frac{\eta^{u}\|e\left(0\right){\|}^{2}-\beta_{2}\frac{\eta_{l}{}^{2}}{\eta^{u}}\chi }{\left(\beta_{1}\beta_{2}\frac{\eta_{l}}{\eta^{u}}-1\right)\chi },\|e\left(0\right)\|>\sqrt{\beta_{2}\frac{\eta_{l}}{\eta^{u}}\chi } \end{array},$$

where $\beta_{2}>\frac{\eta^{u}}{\eta_{l}\beta_{1}}$; hence $\beta_{1}\beta_{2}\frac{\eta_{l}}{\eta^{u}}-1>0$ holds. □

**Remark** **2.** *For the ultimate bound of the tracking error, if Theorem 1 uses Equation (18) as the robust control law, for* $\sigma_{q}\neq 0$ *, the joint position error trajectory will eventually converge to the sliding surface* $\sigma_{q}=0$ *, to obtain a zero steady-state tracking error. In fact, this zero steady-state error is transient, and the trajectory will oscillate near the sliding surface with a certain amplitude. This is caused by measurement errors. This phenomenon can be eliminated by using the following new adaptive control law,*

(52) $$\omega =\{\begin{array}{c} \frac{\rho }{\left\|\sigma_{q}\right\|}\sigma_{q},\;\;if\;\|\sigma_{q}\|\geq \epsilon_{1}\\ \frac{\rho }{\epsilon_{1}}\sigma_{q},\;\;\;\;\;\;if\;\|\sigma_{q}\|<\epsilon_{1} \end{array},$$

*where* $\epsilon_{1}$ *denotes the threshold width of* $\sigma_{q}$ *. This controller cannot ensure that the error converges to zero and that the error changes within a symmetric boundary layer with a thickness of* $2\epsilon_{1}$ *centered on the sliding surface, i.e., the error is UUB. The ultimate bound of Theorem 2 is* r *, which depends on the parameters* $\beta_{0}$ *,* β *and* $\epsilon_{2}$ *. The final tracking error can be reduced by lowering these parameter values, but it also reduces the system’s robustness to noise and external disturbances.*

**Remark** **3.** *The convergence velocity of Theorem 1 is controlled by the parameter* ρ *, while that of Theorem 2 is controlled by* χ *, which is essentially controlled by the parameters* $\beta_{0}$ *,* β *and* $\epsilon_{2}$ *. If these parameters were reduced, the error limit* r *would also be reduced directly, but the time* T *required for the error trajectory to converge to* r *would increase. That is, the convergence velocity will decrease. This can be obtained by the monotonically decreasing property of the second equation in Equation (51) in* χ>0 *.*

**Remark** **4.** *In general, the ultimate bound of error and the convergence velocity of Theorems 1 and 2 can be adjusted by the corresponding parameters. Theorem 2 has many adjustable parameters and hence strong adjustability. In addition, it does not require prior knowledge of the upper bound of uncertainties of the system. However, the design of the control law in Theorem 1 is relatively simple and straightforward, with a small amount of calculation. If* ς *can be obtained accurately, the accuracy of Theorem 1 using Equation (18) will be higher than that of Theorem 2.*

According to the above remarks, the controller should be chosen according to the actual application needs. For example, the action of positioning the charging gun in an automatic charging task requires high accuracy. Therefore, the maximum possible ς should be obtained, and Theorem 1 should be adopted using Equation (18). In another example, a cable-driven neurorehabilitation robot requires a high system response when the robot assists the human arm to move along a certain path. In this case, Theorem 2 can be selected. By reasonably selecting the parameters $\beta_{0}$, β and $\epsilon_{2}$, a balance between rapidity and accuracy is obtained.

### 4.2. Adaptive Robust Control of Motor Torque Outputs

The tracking error of cable input tensions and its filtered tracking error are defined as

(53) $${\tilde{\tau }}_{0}=\tau_{0,d}-\tau_{0}$$

and

(54) $$\sigma_{\tau_{0}}={\dot{\tilde{\tau }}}_{0}+\Lambda_{1}{\tilde{\tau }}_{0},$$

respectively, where $\tau_{0,d}$ is computed by Equation (17), $\tau_{0}$ denotes the actual cable input tensions and can be measured by load cells and $\Lambda_{1}\in {\mathbb{R}}_{m\times m}$ denotes a diagonal positive-definite gain matrix.

Differentiating Equation (8) gives

(55) $$D{\dot{\tau }}_{0}={\dot{\tau }}_{m}-{\dot{\tau }}_{f}.$$

Based on this, the dynamic extension method in [26] can be adopted. Differentiating Equation (61) and then combining it with Equation (55) gives

(56) $$D{\dot{\tilde{\tau }}}_{0}=D{\dot{\tau }}_{0,d}-{\dot{\tau }}_{m}+{\dot{\tau }}_{f}.$$

Combining Equation (55) and Equation (56) gives

(57) $$D\sigma_{\tau_{0}}=D{\dot{\tau }}_{0,d}-{\dot{\tau }}_{m}+{\dot{\tau }}_{f}+D\Lambda_{1}{\tilde{\tau }}_{0}.$$

The following robust control law is adopted:

(58) $${\dot{\tau }}_{m}=D{\dot{\tau }}_{0,d}+{\dot{\hat{\tau }}}_{f}+\zeta ,$$

where $\zeta \in {\mathbb{R}}_{m\times 1}$ denotes the robust integral of the tracking error, which is given by

(59) $$\zeta \left(t\right)=\left(K_{s}+I\right){\tilde{\tau }}_{0}\left(t\right)-\left(K_{s}+I\right){\tilde{\tau }}_{0}\left(0\right)+\int_{0}^{t}\left[\left(K_{s}+I\right)\Lambda_{1}{\tilde{\tau }}_{0}\left(\bar{\sigma }\right)+2K_{r}sgn\left({\tilde{\tau }}_{0}\left(\bar{\sigma }\right)\right)\right]d\bar{\sigma },$$

where $K_{s}\in {\mathbb{R}}_{m\times m}$ and $K_{r}\in {\mathbb{R}}_{m\times m}$ denote gain matrices and sgn(·) denotes the signum function.

Substituting Equation (58) into Equation (57) gives

(60) $$D\sigma_{\tau_{0}}=-{\dot{\hat{\tau }}}_{f}+{\dot{\tau }}_{f}+D\Lambda_{1}{\tilde{\tau }}_{0}-\zeta .$$

Differentiating Equation (60) and then combining it with Equation (59) gives

(61) $$\begin{array}{ll} D{\dot{\sigma }}_{\tau_{0}} & =-{\ddot{\hat{\tau }}}_{f}+{\ddot{\tau }}_{f}+D\Lambda_{1}{\dot{\tilde{\tau }}}_{0}-\dot{\zeta }\\ & =-{\ddot{\hat{\tau }}}_{f}+{\ddot{\tau }}_{f}+D\Lambda_{1}{\dot{\tilde{\tau }}}_{0}-\left(K_{s}+I\right)\sigma_{\tau_{0}}-2K_{r}sgn\left({\tilde{\tau }}_{0}\right)\\ & =-\left(K_{s}+I\right)\sigma_{\tau_{0}}-2K_{r}sgn\left({\tilde{\tau }}_{0}\right)+\tilde{N}+N_{d} \end{array},$$

where $N_{d}=-{\ddot{\hat{\tau }}}_{f}+{\ddot{\tau }}_{f}$, $\tilde{N}=\Lambda_{1}D\left(\sigma_{\tau_{0}}-\Lambda_{1}{\tilde{\tau }}_{0}\right).$

It can be verified that $N_{d}\left(t\right)$, ${\dot{N}}_{d}\in {ℒ}_{\infty }$ by assuming that the first three time derivatives of $\tau_{f}-{\hat{\tau }}_{f}$ are bounded. Furthermore, $\tilde{N}$ can be bounded as

(62) $$\|\tilde{N}{\|}_{2}\leq \varrho \|\delta_{\tau_{0}}{\|}_{2},$$

where $\delta_{\tau_{0}}\in {\mathbb{R}}_{2m\times 2m}$ is defined as $\delta_{\tau_{0}}={\left[\begin{array}{cc} \sigma_{\tau_{0}}^{T} & {\tilde{\tau }}_{0}^{T} \end{array}\right]}^{T}$, $\|\cdot {\|}_{2}$ denotes the Euclidean norm of vector and $\varrho \in {\mathbb{R}}^{+}$ denotes a known positive constant satisfying

(63) $$\varrho \geq \sqrt{2}max\left(\|\Lambda_{1}D{\|}_{F},\|\Lambda_{1}D\Lambda_{1}{\|}_{F}\right),$$

where $\|\cdot {\|}_{F}$ denotes the Frobenius norm of a matrix.

**Theorem** **3.** *Consider the error system defined by Equation (57). The control law described by Equations (58) and (59) and the following sufficient conditions about the adjustable control gains*

(64) $$K_{r}>\|N_{d}{\|}_{\infty }+\Lambda_{1}{}^{-1}\|{\dot{N}}_{d}{\|}_{\infty },\;\;\Lambda_{1}>\frac{1}{2}I_{6},\;\;diag\left\{\lambda_{min}\left(\Lambda_{2}\right)\right\}>\frac{1}{4}\varrho^{2}{\left[\begin{array}{cc} K_{s} & O\\ O & K_{s} \end{array}\right]}^{-1},$$

*ensure a locally exponentially stable result of the tracking errors* ${\tilde{\tau }}_{0}$ *and* $\sigma_{\tau_{0}}$ *, where* $\|\cdot {\|}_{\infty }$ *denotes the infinity norm of a vector and* $\Lambda_{2}\in {\mathbb{R}}_{2m\times 2m}$ *denotes the following defined diagonal positive-definite gain matrix.*

**Proof** **of** **Theorem** **3.** Before introducing the Lyapunov candidate function, we define the auxiliary functions $Q_{\tau_{0}}\left(t\right)$ as

(65) $$Q_{\tau_{0}}\left(t\right)=\int_{0}^{t}2{\dot{\tilde{\tau }}}_{0}{}^{T}K_{r}sgn\left({\tilde{\tau }}_{0}\left(\bar{\sigma }\right)\right)d\bar{\sigma }+2\sum_{i=1}^{m}K_{r,i}\left|{\tilde{\tau }}_{0,i}\left(0\right)\right|-N_{d}^{T}{\tilde{\tau }}_{0}.$$

Using the sufficient condition of the first equation of Equation (64) gives the following inequality:

(66) $$\begin{array}{ll} Q_{\tau_{0}}\left(t\right) & =2\sum_{i=1}^{m}K_{r,i}\left(\left|{\tilde{\tau }}_{0,i}\left(t\right)\right|-\left|{\tilde{\tau }}_{0,i}\left(0\right)\right|\right)+2\sum_{i=1}^{m}K_{r,i}\left|{\tilde{\tau }}_{0,i}\left(0\right)\right|-N_{d}^{T}{\tilde{\tau }}_{0}\\ & =2\sum_{i=1}^{m}K_{r,i}\left|{\tilde{\tau }}_{0,i}\left(t\right)\right|-N_{d}^{T}{\tilde{\tau }}_{0}\\ & \geq 2\sum_{i=1}^{m}\left(\|N_{d}{\|}_{\infty }+\Lambda_{1,i}{}^{-1}\|{\dot{N}}_{d}{\|}_{\infty }\right)\left|{\tilde{\tau }}_{0,i}\left(t\right)\right|-2\sum_{i=1}^{m}\frac{1}{2}\|N_{d}{\|}_{\infty }\left|{\tilde{\tau }}_{0,i}\left(t\right)\right|\\ & =2\sum_{i=1}^{m}\left(\frac{1}{2}\|N_{d}{\|}_{\infty }+\Lambda_{1,i}{}^{-1}\|{\dot{N}}_{d}{\|}_{\infty }\right)\left|{\tilde{\tau }}_{0,i}\left(t\right)\right|\geq 0 \end{array}.$$

The following inequality arises from the second equation in Equation (66):

(67) $$Q_{\tau_{0}}\left(t\right)\leq \sum_{i=1}^{m}\left(2K_{r,i}+\|N_{d}{\|}_{\infty }\right)\left|{\tilde{\tau }}_{0,i}\left(t\right)\right|,$$

where $K_{r,i}$, ${\tilde{\tau }}_{0,i}$ and $\Lambda_{1,i}$ denote the i-th diagonal element of $K_{r}$, ${\tilde{\tau }}_{0}$ and $\Lambda_{1}$, respectively.

Let $V_{\tau_{0}}$ denote the following positive definite function,

(68) $$V_{\tau_{0}}=\frac{1}{2}{\tilde{\tau }}_{0}{}^{T}{\tilde{\tau }}_{0}+\frac{1}{2}\sigma_{\tau_{0}}{}^{T}D\sigma_{\tau_{0}}+Q_{\tau_{0}}\left(t\right).$$

Applying Equations (66) and (67) gives the following lower and upper bounds of $V_{\tau_{0}}$:

(69) $$V_{\tau_{0}}\geq D_{l}{\left(\|\delta_{\tau_{0}}{\|}_{2}\right)}^{2}$$

and

(70) $$V_{\tau_{0}}\leq D^{u}{\left(\|\delta_{\tau_{0}}{\|}_{2}\right)}^{2}+\sum_{i=1}^{m}\left(2K_{r,i}+\|N_{d}{\|}_{\infty }\right)\left|{\tilde{\tau }}_{0,i}\left(t\right)\right|,$$

where $D_{l}=min\left(1/2,D_{ii}\right)\;\mathrm{and}\;D^{u}=max\left(1/2,D_{ii}\right)$ for i=1,2,…,m.

Using Equation (54) multiple times gives the following inequality about the time derivative of $V_{\tau_{0}}$:

(71) $$\begin{array}{ll} {\dot{V}}_{\tau_{0}} & ={\tilde{\tau }}_{0}{}^{T}{\dot{\tilde{\tau }}}_{0}+\sigma_{\tau_{0}}{}^{T}D{\dot{\sigma }}_{\tau_{0}}+{\dot{Q}}_{\tau_{0}}\\ & ={\tilde{\tau }}_{0}{}^{T}{\dot{\tilde{\tau }}}_{0}+\sigma_{\tau_{0}}{}^{T}\left(-\left(K_{s}+I\right)\sigma_{\tau_{0}}-2K_{r}sgn\left({\tilde{\tau }}_{0}\right)+\tilde{N}+N_{d}\right)+2{\dot{\tilde{\tau }}}_{0}{}^{T}K_{r}sgn\left({\tilde{\tau }}_{0}\left(\bar{\sigma }\right)\right)-{\dot{N}}_{d}^{T}{\tilde{\tau }}_{0}-{\dot{\tilde{\tau }}}_{0}{}^{T}N_{d}\\ & ={\tilde{\tau }}_{0}{}^{T}\left(\sigma_{\tau_{0}}-\Lambda_{1}{\tilde{\tau }}_{0}\right)-\sigma_{\tau_{0}}{}^{T}\left(K_{s}+I\right)\sigma_{\tau_{0}}+2\left({\dot{\tilde{\tau }}}_{0}{}^{T}-\sigma_{\tau_{0}}{}^{T}\right)K_{r}sgn\left({\tilde{\tau }}_{0}\right)+\left(\sigma_{\tau_{0}}{}^{T}-{\dot{\tilde{\tau }}}_{0}{}^{T}\right)N_{d}+\sigma_{\tau_{0}}{}^{T}\tilde{N}-{\tilde{\tau }}_{0}{}^{T}{\dot{N}}_{d}\\ & ={\tilde{\tau }}_{0}{}^{T}\sigma_{\tau_{0}}-{\tilde{\tau }}_{0}{}^{T}\Lambda_{1}{\tilde{\tau }}_{0}-\sigma_{\tau_{0}}{}^{T}\left(K_{s}+I\right)\sigma_{\tau_{0}}-2\sum_{i=1}^{m}\Lambda_{1,i}K_{r,i}\left|{\tilde{\tau }}_{0,i}\right|+{\tilde{\tau }}_{0}{}^{T}\Lambda_{1}N_{d}+\sigma_{\tau_{0}}{}^{T}\tilde{N}-{\tilde{\tau }}_{0}{}^{T}{\dot{N}}_{d}\\ & ={\tilde{\tau }}_{0}{}^{T}\sigma_{\tau_{0}}-{\tilde{\tau }}_{0}{}^{T}\Lambda_{1}{\tilde{\tau }}_{0}-\sigma_{\tau_{0}}{}^{T}\left(K_{s}+I\right)\sigma_{\tau_{0}}-\sum_{i=1}^{m}\Lambda_{1,i}K_{r,i}\left|{\tilde{\tau }}_{0,i}\right|+\sigma_{\tau_{0}}{}^{T}\tilde{N}+R_{\tau_{0}} \end{array},$$

where $R_{\tau_{0}}$ is given by

(72) $$R_{\tau_{0}}={\tilde{\tau }}_{0}{}^{T}\Lambda_{1}N_{d}-{\tilde{\tau }}_{0}{}^{T}{\dot{N}}_{d}-\sum_{i=1}^{m}\Lambda_{1,i}K_{r,i}\left|{\tilde{\tau }}_{0,i}\right|.$$

Applying the first equation of Equation (64) gives the following inequality about $R_{\tau_{0}}$:

(73) $$R_{\tau_{0}}\leq {\tilde{\tau }}_{0}{}^{T}\Lambda_{1}N_{d}-{\tilde{\tau }}_{0}{}^{T}{\dot{N}}_{d}-\sum_{i=1}^{m}\Lambda_{1,i}\left(\|N_{d}{\|}_{\infty }+\frac{1}{\Lambda_{1,i}}\|{\dot{N}}_{d}{\|}_{\infty }\right)\left|{\tilde{\tau }}_{0,i}\right|\leq 0.$$

Hence, Equation (71) can be bounded as

(74) $$\begin{array}{ll} {\dot{V}}_{\tau_{0}} & \leq {\tilde{\tau }}_{0}{}^{T}\sigma_{\tau_{0}}-{\tilde{\tau }}_{0}{}^{T}\Lambda_{1}{\tilde{\tau }}_{0}-\sigma_{\tau_{0}}{}^{T}\left(K_{s}+I\right)\sigma_{\tau_{0}}-\sum_{i=1}^{m}\Lambda_{1,i}K_{r,i}\left|{\tilde{\tau }}_{0,i}\right|+\sigma_{\tau_{0}}{}^{T}\tilde{N}\\ & \leq \frac{{\tilde{\tau }}_{0}{}^{T}{\tilde{\tau }}_{0}+\sigma_{\tau_{0}}{}^{T}\sigma_{\tau_{0}}}{2}-{\tilde{\tau }}_{0}{}^{T}\Lambda_{1}{\tilde{\tau }}_{0}-\sigma_{\tau_{0}}{}^{T}\left(K_{s}+I\right)\sigma_{\tau_{0}}-\sum_{i=1}^{m}\Lambda_{1,i}K_{r,i}\left|{\tilde{\tau }}_{0,i}\right|+\sigma_{\tau_{0}}{}^{T}\tilde{N}\\ & =-{\tilde{\tau }}_{0}{}^{T}\left(\Lambda_{1}-\frac{1}{2}I\right){\tilde{\tau }}_{0}-\frac{1}{2}\sigma_{\tau_{0}}{}^{T}\sigma_{\tau_{0}}-\sigma_{\tau_{0}}{}^{T}K_{s}\sigma_{\tau_{0}}-\sum_{i=1}^{m}\Lambda_{1,i}K_{r,i}\left|{\tilde{\tau }}_{0,i}\right|+\sigma_{\tau_{0}}{}^{T}\tilde{N} \end{array},$$

where ${\tilde{\tau }}_{0}{}^{T}\sigma_{\tau_{0}}\leq \left({\tilde{\tau }}_{0}{}^{T}{\tilde{\tau }}_{0}+\sigma_{\tau_{0}}{}^{T}\sigma_{\tau_{0}}\right)/2$ is exploited. Using Equation (62) gives

(75) $$\begin{array}{ll} {\dot{V}}_{\tau_{0}} & \leq -{\tilde{\tau }}_{0}{}^{T}\left(\Lambda_{1}-\frac{1}{2}I\right){\tilde{\tau }}_{0}-\frac{1}{2}\sigma_{\tau_{0}}{}^{T}\sigma_{\tau_{0}}-\sigma_{\tau_{0}}{}^{T}K_{s}\sigma_{\tau_{0}}-\sum_{i=1}^{m}\Lambda_{1,i}K_{r,i}\left|{\tilde{\tau }}_{0,i}\right|+\varrho \|\sigma_{\tau_{0}}{\|}_{2}\|\delta_{\tau_{0}}{\|}_{2}\\ & \leq -\lambda_{min}\left(\Lambda_{2}\right)\delta_{\tau_{0}}{}^{T}\delta_{\tau_{0}}-\sigma_{\tau_{0}}{}^{T}K_{s}\sigma_{\tau_{0}}-\sum_{i=1}^{m}\Lambda_{1,i}K_{r,i}\left|{\tilde{\tau }}_{0,i}\right|+\varrho \|\sigma_{\tau_{0}}{\|}_{2}\|\delta_{\tau_{0}}{\|}_{2}\leq -\delta_{\tau_{0}}{}^{T}\Lambda_{3}\delta_{\tau_{0}}-\sum_{i=1}^{m}\Lambda_{1,i}K_{r,i}\left|{\tilde{\tau }}_{0,i}\right| \end{array},$$

where the two inequalities ${\tilde{\tau }}_{0}{}^{T}\left(\Lambda_{1}-\frac{1}{2}I\right){\tilde{\tau }}_{0}+\frac{1}{2}\sigma_{\tau_{0}}{}^{T}\sigma_{\tau_{0}}=\delta_{\tau_{0}}{}^{T}\Lambda_{2}\delta_{\tau_{0}}\geq \lambda_{min}\left(\Lambda_{2}\right)\delta_{\tau_{0}}{}^{T}\delta_{\tau_{0}}$ and $\lambda_{min}\left(\Lambda_{2}\right)\delta_{\tau_{0}}{}^{T}\delta_{\tau_{0}}+\sigma_{\tau_{0}}{}^{T}K_{s}\sigma_{\tau_{0}}-\varrho \|\sigma_{\tau_{0}}{\|}_{2}\|\delta_{\tau_{0}}{\|}_{2}\geq \delta_{\tau_{0}}{}^{T}\Lambda_{3}\delta_{\tau_{0}}$ are exploited and $\Lambda_{2}\in {\mathbb{R}}_{2m\times 2m}$ and $\Lambda_{3}\in {\mathbb{R}}_{2m\times 2m}$ are given by

(76) $$\Lambda_{2}=\left[\begin{array}{cc} \frac{1}{2}I & O\\ O & \Lambda_{1}-\frac{1}{2}I \end{array}\right],\;\Lambda_{3}=diag\left\{\lambda_{min}\left(\Lambda_{2}\right)\right\}-\frac{1}{4}\varrho^{2}{\left[\begin{array}{cc} K_{s} & O\\ O & K_{s} \end{array}\right]}^{-1}.$$

Here, the following inequalities about $\Lambda_{2}\;\mathrm{and}\;\Lambda_{3}$

(77) $$\lambda_{min}\left(\Lambda_{2}\right)>0,\;\Lambda_{3}>O$$

are required to ensure the following proof process, which can naturally derive the second and third equations of Equation (64). Hence, by using Equation (70), the following inequality is easily derived:

(78) $$\begin{array}{ll} {\dot{V}}_{\tau_{0}} & \leq -\delta_{\tau_{0}}{}^{T}\Lambda_{3}\delta_{\tau_{0}}-\sum_{i=1}^{m}\Lambda_{1,i}K_{r,i}\left|{\tilde{\tau }}_{0,i}\right|\leq -\alpha_{3}{\left(\left\|\delta_{2}\right\|\right)}^{2}-\sum_{i=1}^{m}\Lambda_{1,i}K_{r,i}\left|{\tilde{\tau }}_{0,i}\right|\\ & =\frac{\alpha_{3}}{D^{u}}D^{u}{\left(\|\delta {\|}_{2}\right)}^{2}+\frac{\Lambda_{1,i}K_{r,i}}{2K_{r,i}+\|N_{d}{\|}_{\infty }}\sum_{i=1}^{m}\left(2K_{r,i}+\|N_{d}{\|}_{\infty }\right)\left|{\tilde{\tau }}_{0,i}\left(t\right)\right|\\ & \leq -\alpha_{4}\left(D^{u}{\left(\|\delta {\|}_{2}\right)}^{2}+\sum_{i=1}^{m}\left(2K_{r,i}+\|N_{d}{\|}_{\infty }\right)\left|{\tilde{\tau }}_{0,i}\left(t\right)\right|\right)\leq -\alpha_{4}V_{\tau_{0}} \end{array},$$

where $\alpha_{3}=\lambda_{min}\left(\Lambda_{3}\right)$ and $\alpha_{4}$ can be chosen as

(79) $$0<\alpha_{4}<min\left(\alpha_{3}/D^{u},\;\left(\Lambda_{1,i}K_{r,i}\right)/\left(2K_{r,i}+\|N_{d}{\|}_{\infty }\right)\right)$$

for i=1,2,…,m.

Therefore, the error ${\tilde{\tau }}_{0}$ and $\sigma_{\tau_{0}}$ are exponentially stable. □

**Remark** **5.** *Theorems 1 and 2 give the global asymptotic stability and UUB stability of the joint-position-tracking error* $\sigma_{q},\;\tilde{q}$ *, respectively. Theorem 3 gives the local exponential stability of the cable-tension-tracking error* $\sigma_{\tau_{0}},{\tilde{\tau }}_{0}$ *.*

## 5. Stability Analysis of Cascaded System

The existence of the coupling term $f_{\Delta }$ in Equation (10) makes it impossible to always obtain the stability of the entire closed-loop system based on the combination of Theorem 1 and Theorem 3 or Theorem 2 and Theorem 3 forming the robust adaptive controllers with a known upper bound and without an upper bound of uncertainties, respectively, which only ensures the stability of the high-level and low-level subsystems. However, the stability of the entire cascade system has yet to be proven.

**Lemma** **1.** *Based on Theorem 3, the low-level subsystem* ${\dot{e}}_{\tau_{0}}=f_{e_{\tau_{0}}}\left(e_{\tau_{0}},{\ddot{\tau }}_{m},{\ddot{\tau }}_{0,d},{\ddot{\tau }}_{f}\right)$ *is locally exponentially stable. Based on Theorem 1 or Theorem 2, the high-level subsystem ignoring the coupling term* $f_{\Delta }$ *,* ${\dot{e}}_{q}=f_{e_{q}}\left(e_{q},\mu ,q,\dot{q},\ddot{q}\right)$ *is globally asymptotically stable or UUB. Hence, the cascaded system described by Equation (10) is asymptotically stable if its solutions are all uniformly bounded.*

**Proof** **of** **Lemma** **1.** The proof is already given by [27] and [28]. □

**Lemma** **2.** *Combining this lemma with Theorem 3, all solutions of the cascaded system described by Equation (10) are all uniformly bounded [29,30], provided that*

*(a) The coupling term* $f_{\Delta }$ *is bounded as*

(80) $$\|f_{\Delta }\left(e_{q},e_{\tau_{0}}\right)\|\leq \varphi_{2}\left(\|e_{\tau_{0}}\|\right)\|e_{q}\|+\varphi_{1}\left(\|e_{\tau_{0}}\|\right),$$

*where* $\varphi_{1}$ *and* $\varphi_{2}$ *are class* K *functions.*

*(b) There exist positive constants* $k_{1}$ *and* $k_{2}$ *, such that for* $\|e_{q}\|\geq k_{2},$ *the two Lyapunov functions* V *in Theorems 1 and 2, which establish the stability of* ${\dot{e}}_{q}=f_{e_{q}}\left(e_{q},\mu ,q,\dot{q},\ddot{q}\right)$ *, satisfy*

(81) $$\|\frac{\partial V}{\partial e_{q}}\|\|e_{q}\|\leq k_{1}V.$$

**Proof** **of** **Lemma** **2.** According to $\mu =J\tau_{0,d}$ and ${\tilde{\tau }}_{0}=S_{3}e_{\tau_{0}}$, the norm of $f_{\Delta }$ can be given by

(82) $$\begin{array}{ll} \|f_{\Delta }\left(e_{q},e_{\tau_{0}})\right\| & =\|S_{2}M^{-1}\left(J{\tilde{\tau }}_{0}-J\tau_{0,d}+\mu \right)\|=\|S_{2}M^{-1}\left(J{\tilde{\tau }}_{0}-J\tau_{0,d}+J\tau_{0,d})\right\|\\ & =\|S_{2}M^{-1}J{\tilde{\tau }}_{0}\|=\|S_{2}M^{-1}JS_{3}e_{\tau_{0}}\|\leq \|Q\|\|e_{\tau_{0}}\|=\varphi_{1}\left(\|e_{\tau_{0}}\|\right) \end{array},$$

where $S_{3}=\left[\begin{array}{cc} I_{n\times n} & O_{n\times n} \end{array}\right]$, $Q=S_{2}M^{-1}JS_{3}$, $\varphi_{1}\in K$. The class K function $\varphi_{2}$ in Equation (80) does not exist because that $f_{\Delta }$ is not a function of $e_{q}$. Hence, Equation (82) supports condition (a).

The tracking errors $\tilde{q}$ and $\sigma_{q}$ can be written as functions of $e_{q}$:

(83) $$\tilde{q}=S_{3}e_{q},\sigma_{q}=\left(S_{4}+\Lambda S_{3}\right)e_{q},$$

where $S_{4}=\left[\begin{array}{cc} O_{n\times n} & I_{n\times n} \end{array}\right]$. Substituting Equation (83) into V involved in Theorem 1 gives

(84) $$\begin{array}{ll} V & =\frac{1}{2}\sigma_{q}{}^{T}M\left(q\right)\sigma_{q}+{\tilde{q}}^{T}\Lambda K_{D}\tilde{q}+\frac{1}{2}{\tilde{\pi }}^{T}K_{\pi }\tilde{\pi }\\ & =\frac{1}{2}{\left[\left(S_{4}+\Lambda S_{3}\right)e_{q}\right]}^{T}M\left(q\right)\left[\left(S_{4}+\Lambda S_{3}\right)e_{q}\right]+{\left(S_{3}e_{q}\right)}^{T}\Lambda K_{D}\left(S_{3}e_{q}\right)+\frac{1}{2}{\tilde{\pi }}^{T}K_{\pi }\tilde{\pi }\\ & =e_{q}{}^{T}Se_{q}+\frac{1}{2}{\tilde{\pi }}^{T}K_{\pi }\tilde{\pi }\geq e_{q}{}^{T}Se_{q} \end{array},$$

where $S={\left(S_{4}+\Lambda S_{3}\right)}^{T}M\left(q\right)\left(S_{4}+\Lambda S_{3}\right)+S_{3}{}^{T}\Lambda K_{D}S_{3}$. The inequality of Equation (84) also applies to V in Theorem 2.

Therefore, there exist positive constants $k_{1}$ and $k_{2}$, which satisfy

(85) $$\|\frac{\partial V}{\partial e_{q}}\|\|e_{q}\|=\left\|(S+S^{T}\right)e_{q}\|\|e_{q}\|\leq 2\|S{\|}_{2}\|e_{q}{\|}^{2}\leq k_{1}V$$

with $\|e_{q}\|>k_{2}$. The inequality of Equation (85) also applies to V in Theorem 2. Hence, Equation (85) supports condition (b). □

**Theorem** **4.** *Given the controller design described by Equations (15), (17) and (58), the robust control law described by Equation (18) and the adaptive update law described by Equation (19) or the controller design described by Equations (15), (17) and (58), the robust control law described by Equation (20) and the adaptive update law described by Equations (21) and (22), the closed-loop system Equation (10) is in asymptotically stable equilibrium at* $\left(e_{q},e_{\tau_{0}}\right)=\left(0,0\right)$ *.*

**Proof** **of** **Theorem** **4.** Obviously, the combination of Theorems 1 and 3 or Theorems 2 and 3 together with Lemmas 1 and 2 ensures the asymptotic stability of the equilibrium point $\left(e_{q},e_{\tau_{0}}\right)=\left(0,0\right)$. □

**Figure 3:**
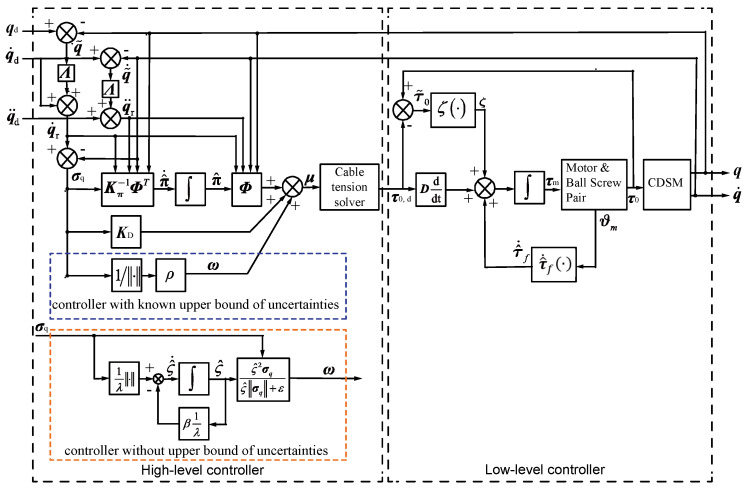
Diagram of the proposed robust adaptive controllers with a known upper bound and without an upper bound of uncertainties.

The block diagrams of the controllers with a known upper bound and without an upper bound of uncertainties are shown in Figure 3.

## 6. Experiment

### 6.1. Experimental Setup

**Figure 4:**
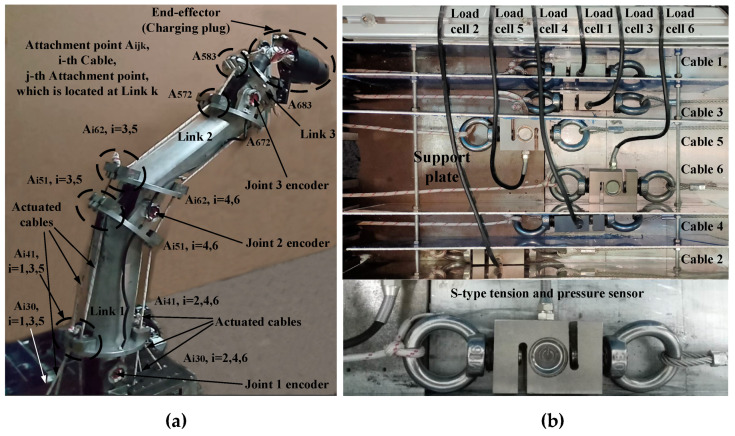
Prototype of (**a**) a three-DOF six-cable CDSM and (**b**) system prototype for cable tension measurement.

To verify the adaptability of the proposed controller to actual application scenarios, a three-DOF six-cable CDSM for an automatic charging robot of electric vehicles is used as the research object of this experiment to verify whether the controller can complete the actual task of automatic charging, as shown in Figure 4a. The CDSM has three links and three rotational DOFs corresponding to the three links. The charging plug is connected to Link 3 as an end-effector. Hence, the DOF information can be given by $n_{1}=n_{2}=n_{3}=1$ and n=3. Besides, the revolute axes are parallel to each other. The vector of joint space can be represented as $q={\left[\begin{array}{ccc} \theta_{1} & \theta_{2} & \theta_{3} \end{array}\right]}^{T}\in {\mathbb{R}}_{3\times 1}$. The joint positions can be measured by joint encoders, which are mounted on the joint axes. The cable system actuating the CDSM consists of 6 cables with a total 36 attachment points and 30 cable segments, i.e., m=6 and $n_{s}=30$.

**Figure 5:**
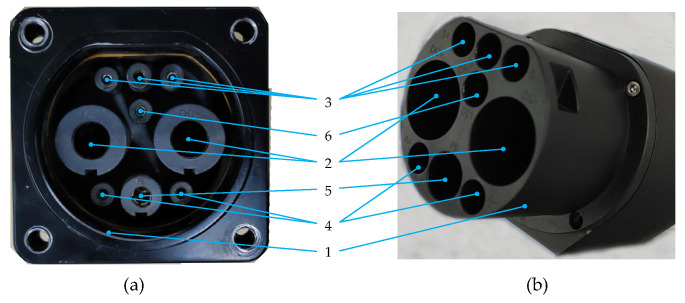
The (**a**) charging port and (**b**) charging plug in the automatic charging scenario of electric vehicles.

Automatic charging robots for electric vehicles are a research field which has emerged in recent years [5,31,32]. Its purpose is to use robots to transform manual process plugging charging plugs into charging ports to an automatic plugging-unplugging operation, as shown in Figure 5. In the process of plugging and unplugging, there are six different peg-in-hole assemblies according to the minimum clearances between pegs and holes, where the first one (#1) is the charging gun’s outer diameter and the charging port’s inner diameter, and the remaining five (#2, 3, 4, 5 and 6) are the contact’s outer diameters and the jack’s inner diameters.

The realisation of this kind of automation will free the charging workers from heavy and repetitive work. A complete automatic charging process is shown in Figure 7 in [5]. The task of the controller proposed in this paper is to ensure the movement accuracy of the two sub-processes from the starting position to the pre-plugging position and from the pre-plugging position to the plugging position.

The CDSM system was equipped with three types of sensors: motor encoders, joint encoders capable of providing accurate position measurements for the joints and load cells capable of yielding accurate cable tensions. The load cell is selected as an S-type tension and pressure sensor. To reduce the influence of the sensor’s weight on the cable tension measurement, the load cell was installed horizontally, so its weight could be supported by the upper surface of the support plate (see Figure 4b). A sufficient range of motion was provided for the load cells so that there would be no interference between the load cells and the pulleys on both sides of them, ensuring that the predetermined range of motion of the robot was reachable. The data acquisition system of cable tension consists of a high-precision weight transmitter, an NI cDAQ-9179 data acquisition platform and an NI 9923 connector block. The sampling time can reach 4 ms. Given that the sampling time of the joint encoders is also 4 ms, the control cycle of the entire control system is 4 ms, which is sufficient for real-time execution of the designed controller.

For this three-DOF six-cable robot, detailed information about the Jacobian matrices V, W and L in Equation (3) and the matrix Φ and parameter vector π in Equation (15) are provided in Appendix A and Appendix B, respectively.

The parameters of the robot and the ball-screw system are listed in Table 2, where the symbols $C_{k}$ and $m_{k}$ (k=1,⋯,p) denote the center of mass and the mass of Link k, respectively, and the symbols $r_{k,C_{k},x}^{k}$, $r_{k,C_{k},y}^{k}$ and $r_{k,C_{k},z}^{k}$ denote the first, second and third elements of the position vector $r_{k,C_{k}}^{k}$ of the origin of Frame $O_{k}$ and the center of mass $C_{k}$, respectively.

To verify the effectiveness of the controllers with a known upper bound and without an upper bound of uncertainties, two sets of experiments were performed with the three-DOF six-cable CDSM system.

### 6.2. Experimental Results

#### 6.2.1. Path and Trajectory to Be Tracked

**Figure 6:**
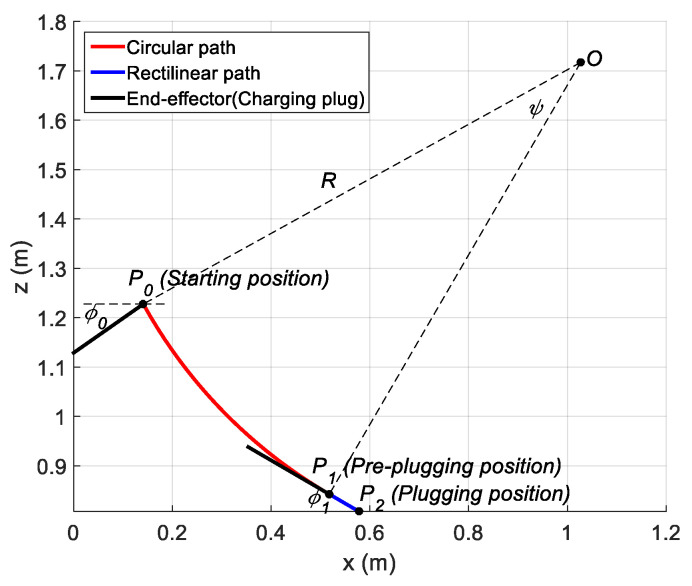
Planned path containing a circular arc and a rectilinear for the auto-charging task of electric vehicles.

In this experiment, a path containing a circular arc $P_{0}P_{1}$ with radius R=1.012 m and central angle ψ=0.5393 rad and a rectilinear path $P_{1}P_{2}$ at orientation $\phi_{1}=0.5271$ rad were planned for the robot end-effector (charging plug). The rectilinear sub-path is tangential to the circular sub-path (see Figure 6). In the automatic charging scenario, points $P_{0}$, $P_{1}$ and $P_{2}$ denote the starting, pre-plugging and plugging positions, respectively. The angle $\phi_{1}$ denotes the angle between the central axis of the charging port and the horizontal plane. The angle $\phi_{0}$ denotes the angle between the central axis of the charging plug and the horizontal plane.

For the position trajectory of the circular sub-path, a cubic trajectory was planned for the arc length first, which is defined as the length of the arc with extremes P and $P_{0}$, where P is a generic point in $P_{0}P_{1}$. The position trajectory was easily obtained by parametrically representing the circular path as a function of the arc length. Similarly, the position trajectory can be planned according to the trapezoidal velocity profile for the rectilinear sub-path. For the orientation trajectory of the circular sub-path, a cubic trajectory was still chosen, where the initial and final values were $\phi_{0}=-0.6109$ rad and $\phi_{1}$, respectively. For the rectilinear sub-path, the orientation of the end-effector (charging plug) maintained $\phi =\phi_{1}$ at all times during the motion of the rectilinear sub-path.

The trajectory of the operation space can be converted into that of the joint space by using inverse kinematics. In this experiment, the method of tracking the joint angle trajectory was adopted to ensure the trajectory of the end-effector.

#### 6.2.2. Results of the Controller with a Known Upper Bound of Uncertainties

**Figure 7:**
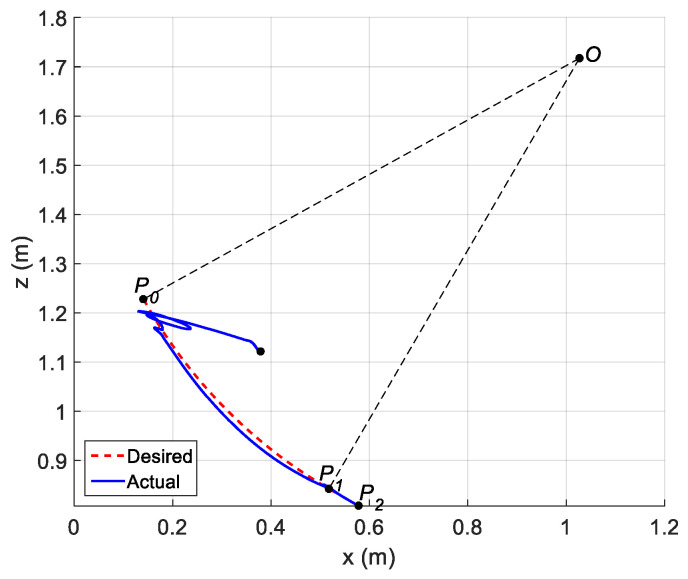
Experiment for the proposed robust adaptive controller with a known upper bound of uncertainties: desired and actual paths.

**Figure 8:**
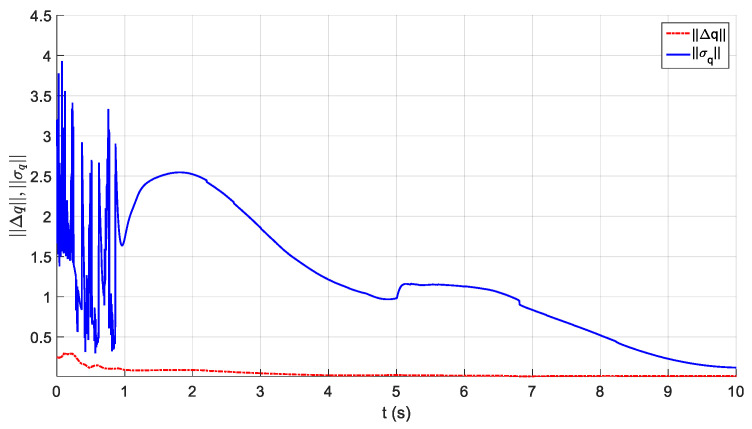
Experiment for the proposed robust adaptive controller with a known upper bound of uncertainties: norm of the joint-tracking error.

**Figure 9:**
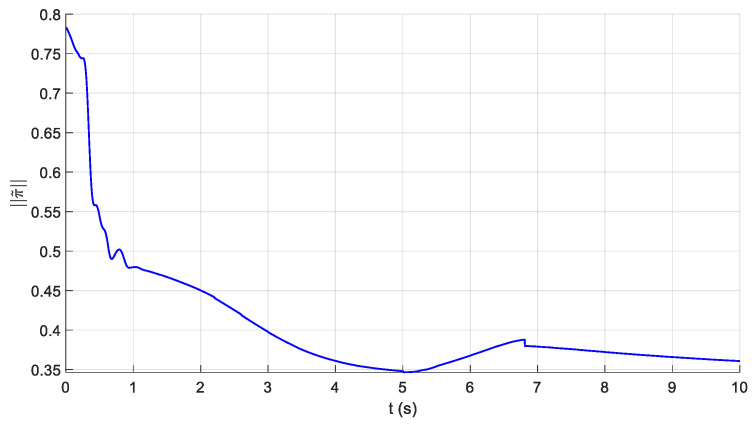
Experiment for the proposed robust adaptive controller with a known upper bound of uncertainties: time evolution of the norm updates of the parameter error $\tilde{\pi }$

**Figure 10:**
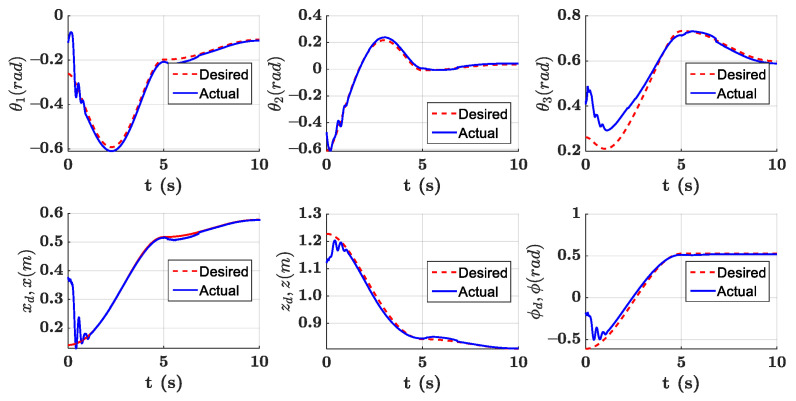
Experiment for the proposed robust adaptive controller with a known upper bound of uncertainties: desired and actual trajectories in operational and joint spaces.

**Figure 11:**
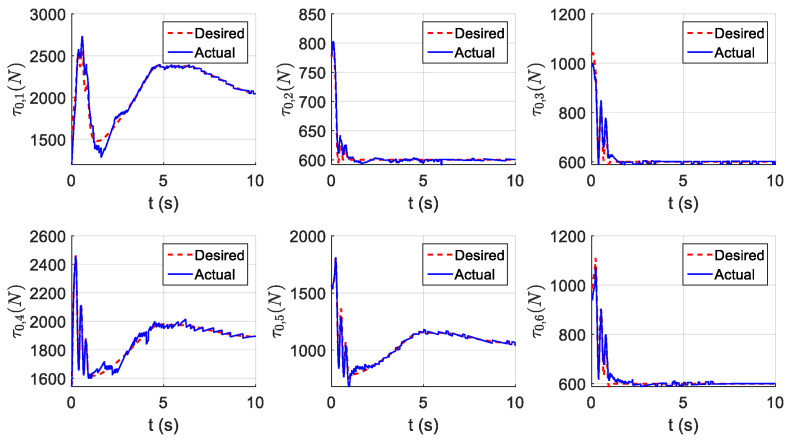
Experiment for the proposed robust adaptive controller with a known upper bound of uncertainties: planned and actual cable tension trajectories.

The parameters of the controller from the first experiment are given in Table 3. The initial position of the CDSM in the circular sub-path was set at $q\left(0\right)=q_{d}\left(0\right)+{\left[0.15\;0.15\;0.15\right]}^{T}$. The initial position of the rectilinear sub-path is the tracking result of the end position of the previous sub-path. The initial value of adaptive parameters was set at $\hat{\pi }\left(0\right)=0.9\pi$. The experimental results are given in Figure 7, Figure 8, Figure 9, Figure 10 and Figure 11. The desired and actual paths are given in Figure 7. The norm of the joint-tracking error is shown in Figure 8, and the time evolution of the norm updates of dynamic parameter error $\left\|\tilde{\pi }\right\|$ is illustrated in Figure 9. The desired and actual trajectories in the joint and operational spaces are demonstrated in Figure 10. The corresponding cable tension trajectories are shown in Figure 11.

Figure 8 reveals that the tracking error of joint angle remains within a small bounded area when the high-level controller based on Theorem 1 is used. Simultaneously, Figure 11 shows that the cable tensions remain positive and are well tracked when the low-level controller based on Theorem 3 is used. These results verify the validity and effectiveness of the proposed controllers based on the combination of Theorems 1 and 3. Nonetheless, the adaptive parameters do not converge to their nominal values, as confirmed by the time history of the norm of the parameter error vector which reaches a non-null steady-state value (see Figure 9).

#### 6.2.3. Results of the Controller without an Upper Bound of Uncertainties

**Figure 12:**
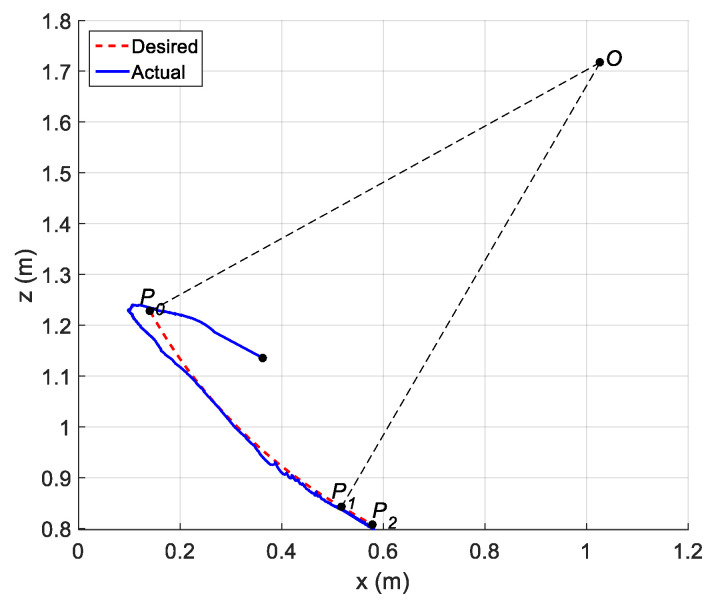
Experiment for the proposed robust adaptive controller without an upper bound of uncertainties: desired and actual paths.

**Figure 13:**
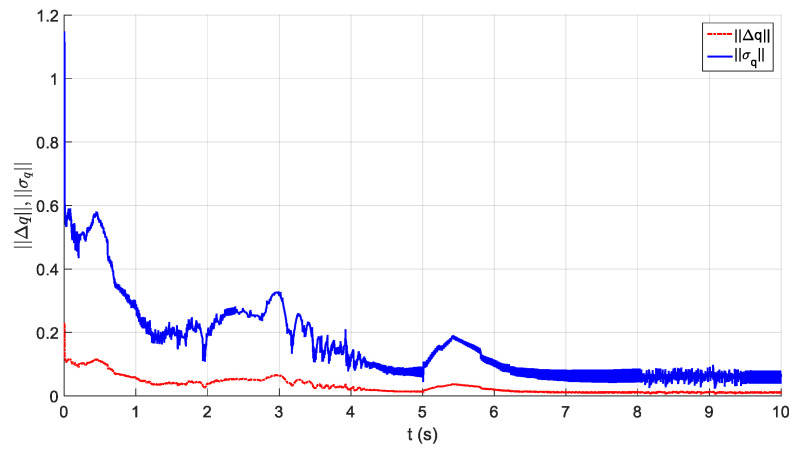
Experiment for the proposed robust adaptive controller without an upper bound of uncertainties: norm of the joint-tracking error.

**Figure 14:**
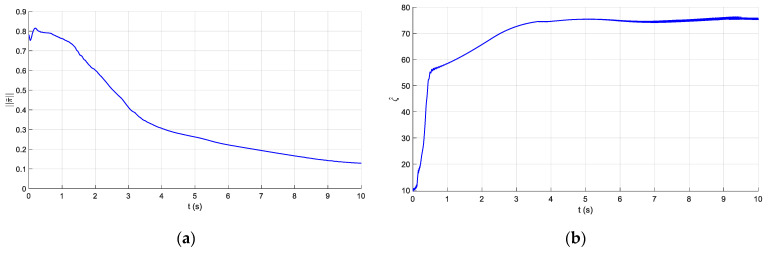
Experiment for the proposed robust adaptive controller without an upper bound of uncertainties: time evolution of parameter updates of $\tilde{\pi }$ and $\hat{\varsigma }$. (**a**) Time evolution of norm updates of the parameter error $\left\|\tilde{\pi }\right\|$ and (**b**) time evolution of parameter updates of $\hat{\varsigma }$.

**Figure 15:**
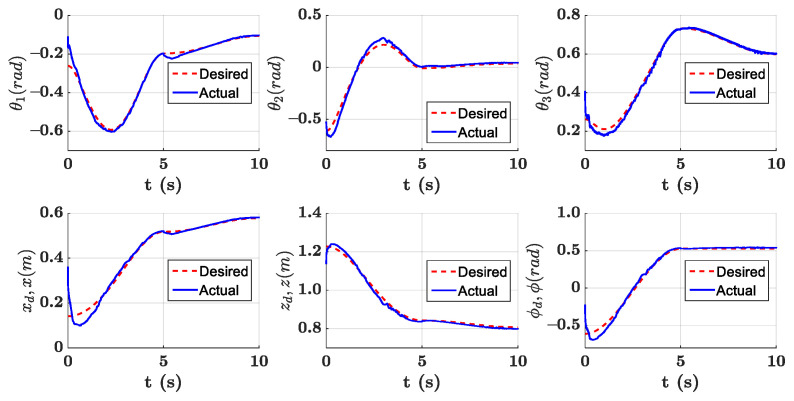
Experiment for the proposed robust adaptive controller without an upper bound of uncertainties: desired and actual trajectories in operational and joint spaces.

**Figure 16:**
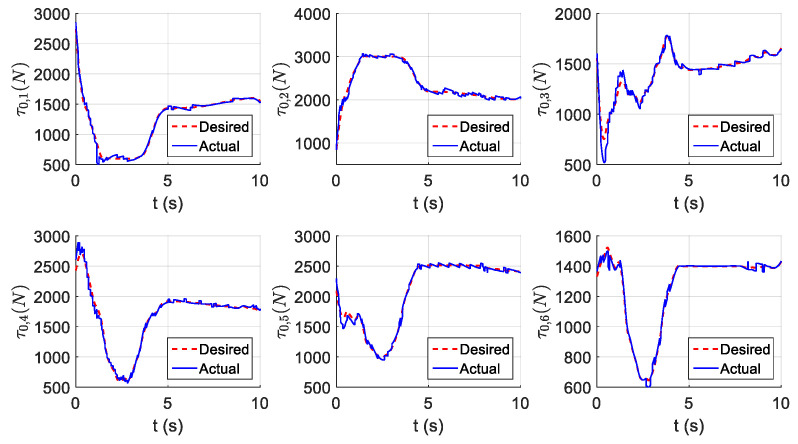
Experiment for the proposed robust adaptive controller without an upper bound of uncertainties: planned and actual cable tension trajectories.

The parameters of the controller from the second experiment are listed in Table 4, where all diagonal elements of Λ have values between 0.5 and 1 to ensure $K_{D}$ is independent of the unknown viscous friction coefficient $F_{ii}$. The parameters of the lower-level controller are identical to those in the first experiment. The initial positions of the robot in the circular sub-path and the rectilinear sub-path and the initial values of the adaptive parameters are identical to those in the first experiment. The experimental results are given in Figure 12, Figure 13, Figure 14, Figure 15 and Figure 16. As was observed from this experiment, the tracking error of the joint angle remained within a small bounded area when the proposed high-level controller based on Theorem 2 was used (see Figure 13). Moreover, the cable tensions remained positive and were well tracked when the proposed low-level controller based on Theorem 3 was used (see Figure 16). These results verify the validity and effectiveness of the proposed controllers based on the combination of Theorems 2 and 3. Besides, Figure 14b shows that the estimation of the upper bound of uncertainties reaches near a non-null steady-state value. Nevertheless, this value is not necessarily its true value.

Comparing the first and second experiments, the following results were obtained. First, the initial trajectory tracking of 0 to 1 s of the second experiment is more stable than that of the first experiment, when comparing Figure 8 and Figure 13. However, the joint-tracking error of the second experiment increased significantly in the initial stage of the rectilinear sub-trajectory, i.e., 5 to 6 s, which does not appear in the first experiment. This indicates that the high-level controller based on Theorem 1 has a stronger ability to adapt to the trajectories with discontinuous acceleration than the high-level controller based on Theorem 2. These results show that the two types of high-level controller have their own advantages and disadvantages. Accordingly, different controllers should be chosen for different application scenarios. For tasks which require high positional accuracy but low intermediate track-tracking accuracy, the controller based on the combination of Theorems 1 and 3 should be used. For tasks which require high trajectory-tracking accuracy, the controller based on the combination of Theorems 2 and 3 should be selected. The trajectory-tracking and positioning accuracies of the above experimental results meet the requirements of the automatic charging task of electric vehicles.

#### 6.2.4. Results of the PID Controller

**Figure 17:**
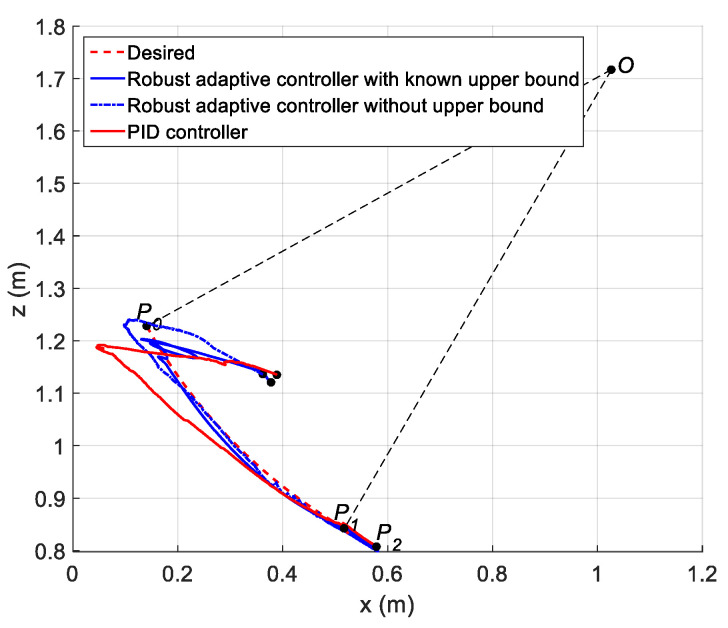
Experiment for the proportional–integral–derivative (PID) controller: desired and actual paths.

**Figure 18:**
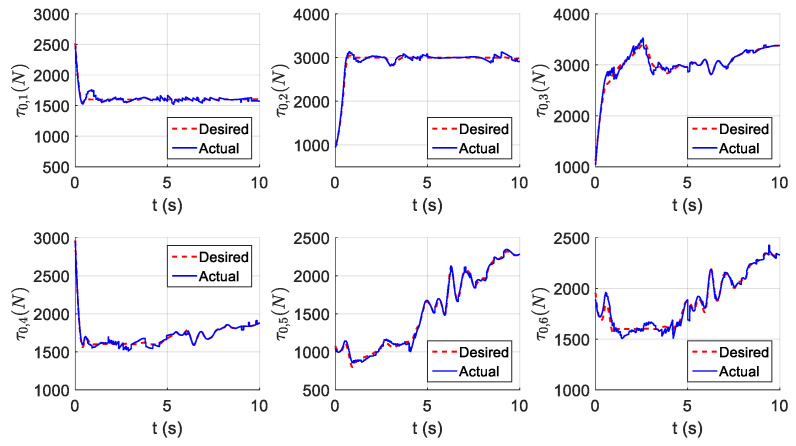
Experiment for the PID controller: planned and actual cable tension trajectories.

To demonstrate the superiority of both proposed controllers with a known upper bound and without an upper bound of uncertainties over the classical PID controller, some comparison results are presented here. First, having the same experiment conditions as the previous study, the high-level controller is replaced with a well-tuned PID controller, which is commonly used in the literature for the tracking of the desired joint position. The desired and actual paths, using the three controllers, are illustrated and compared in Figure 17. The cable tension trajectories using the PID controller are shown in Figure 18.

Compared with the classical PID controller, the two proposed controllers have significant improvements according to Figure 17. In addition, compared with the two proposed controllers, the cable tensions generated by the PID controller are larger, which means that the auto-charging system using the CDSM consumes more energy. It should be noted that the three starting points of the paths corresponding to the three controllers in Figure 17 are very close but not completely coincident. In fact, this is normal and allowed, which is because the three experiments are carried out separately, so it is impossible for the CDSM to have exactly the same posture at the initial moment of each experiment.

## 7. Conclusions

This study proposes a robust adaptive controller for stable tracking of the joint position and cable tension trajectories in a CDSM. First, two robust adaptive controllers were designed for the task of joint position trajectory tracking in two scenarios, i.e., with known and unknown upper bounds of uncertainties, respectively. The stabilities, ultimate bounds of tracking error and convergence speeds of the two high-level controllers were compared theoretically in remarks. Compared with the adaptive controllers for a CDPM in existing research, the two controllers in this study included the joint viscous friction coefficient in their dynamic parameter vectors, which is a factor which must be considered for CDSMs’ controller design. Besides, an important advantage of the controller in Theorem 2 is that it makes the control gains independent of the upper bound of uncertainties, which is superior to existing CDPMs’ adaptive controllers. Second, for the task of cable tension tracking, this paper proposes a robust controller based on the dynamics of a ball-screw system. By using the Lyapunov method, we analysed the stability of the part of the subsystem from the cable tension to the joint position, the part of the subsystem from the motor output torque to the cable tension and the entire system. Experiments with a three-DOF six-cable CDSM demonstrated the validity and effectiveness of the designed controller. The comparison experiment with the classical PID controller verified the superiority of both proposed controllers with a known upper bound and without an upper bound of uncertainties. Further works should focus on improving the proposed controllers’ adaptability to hyper-redundant CDSMs, the link and cable numbers of which are as large as tens. This is because the hyper-redundancy will cause bulky work of the control gains’ selection when the proposed controllers are used. Moreover, the controllers proposed in this paper only consider viscous friction, although good experimental results have been obtained. However, if Coulomb friction is also taken into consideration, whether better experimental results can be obtained remains to be verified. This is also future research work.

## Figures and Tables

**Table 1:** Mathematical symbols and their physical meanings used in the controller design.

Math Symbol	Physical Meaning	Comment
M	Inertia matrix	Dynamic parameters of the CDSM
C	Centrifugal and Coriolis matrices	
F	Diagonal matrix of viscous friction coefficients	
g	Vector of the gravity term	
V	Jacobian matrix relating τ to $\omega_{T}$	Jacobian matrices
W	$\mathrm{Jacobian}\;\mathrm{matrix}\;\mathrm{relating}\;\omega_{e}$ to joint moments	
L	$\mathrm{Matrix}\;\mathrm{relating}\;\tau_{0}$ and τ	
J	$\mathrm{Jacobian}\;\mathrm{matrix}\;\mathrm{relating}\;\tau_{0}$ to joint moments	
Φ	Regression matrix	Linearity in the dynamic parameters
π	Vector of constant dynamic parameters	
$q,\dot{q},\ddot{q}$	Vector of joint position, velocity, acceleration	$\mathrm{Desired}\;\mathrm{joint}\;\mathrm{trajectory}\;q_{d}$ tracked by high-level controller
$\tau_{0}$	Vector of the input cable tensions	$\mathrm{Desired}\;\mathrm{input}\;\mathrm{cable}\;\mathrm{tensions}\;\tau_{0,d}$ tracked by low-level controller
$\tau_{m}$	Vector of motor torque outputs	Controller design object of low-level controller in Equation (58)
u	Control input of joint moment	Controller design in Equation (15)
$e_{q},e_{\tau_{0}}$	Tracking errors of joint positions, input cable tensions	-
Λ	Parameters of the high-level controller with a known upper bound of uncertainties. They meet the condition of Equation (23).	In Equation (16)
$K_{D}$		In Equation (15)
$K_{\pi }$		In Equation (19)
ρ		In Equation (18)
$\epsilon_{1}$		In Equation (52)
Λ	Parameters of the high-level controller without an upper bound of uncertainties. They meet the condition of Equation (24).	In Equation (16)
$K_{D}$		In Equation (15)
$K_{\pi },\beta_{0}$		In Equation (21)
λ,β		In Equation (22)
$\epsilon_{2}$		In Equation (20)
$\Lambda_{1}$	Low-level controller parameters which meet the condition of Equation (64)	In Equation (54)
$K_{s},K_{r}$		In Equation (59)

**Table 2:** Parameters of the robot and ball-screw system.

Parameter	Value	Unit
$m_{1},m_{2},m_{3}$	5.0581, 4.7451, 3.4377	kg
$I_{1,yy}^{1},I_{2,yy}^{2},I_{3,yy}^{3}$	0.067485, 0.074783, 0.025347	kg·m^2^
$r_{1,C_{1}}^{1}$	${\left[\begin{array}{ccc} 0.069546 & 0 & -0.15156 \end{array}\right]}^{T}$	m
$r_{2,C_{2}}^{2}$	${\left[\begin{array}{ccc} -0.073136 & 0.0010443 & -0.15226 \end{array}\right]}^{T}$	m
$r_{3,C_{3}}^{3}$	${\left[\begin{array}{ccc} 0.055926 & -0.0044049 & -0.037974 \end{array}\right]}^{T}$	m
D	$0.6366\times 10^{-3}I_{6}$	m/rad
$J_{m}$	$9.6\times 10^{-6}I_{6}$	kg·m^2^
$J_{s}$	$6.8\times 10^{-6}I_{6}$	kg·m^2^
$M_{t}$	$0.386I_{6}$	kg

**Table 3:** Experiment parameters for the robust adaptive controller with a known upper bound of uncertainties.

Parameter	Value	Unit
Λ	5$I_{3}$	-
$K_{D}$	diag([5 10 5])	-
$K_{\pi }$	diag([2E3 1E5 5E4 5E5 5E5 5E5 5E5 9E5 9E4 9E6 5E6 9E6 3E4])	-
ρ	130	-
$\epsilon_{1}$	0.04	-
$\hat{\pi }\left(0\right)$	0.9π	Same as π
$\Lambda_{1}$	$I_{6}$	-
$K_{s}$	$3I_{6}$	-
$K_{r}$	$2I_{6}$	-

**Table 4:** Experiment parameters for the proposed robust adaptive controller without an upper bound of uncertainties.

Parameter	Value	Unit
Λ	0.8$I_{3}$	-
$K_{D}$	5$I_{3}$	-
$K_{\pi }$	diag([2E1 1E5 0.5E4 5E2 5E1 1E5 1.0E4 5E4 3E2 1E5 1E5 5E5 2E2])	-
λ	0.5	-
$\beta_{0}$	0.01	-
β	0.01	-
$\epsilon_{2}$	0.04	-
$\hat{\pi }\left(0\right)$	0.9π	Same as π
$\hat{\varsigma }\left(0\right)$	15	Nm

## Data Availability

The code of the controller proposed in this paper is provided. It is written in C language and is attached to the framework of the POWERLINK communication protocol.

## Appendix A

The appendix is an optional section which can contain details and data supplemental to the main text, for example, explanations of experimental details which would disrupt the flow of the main text but nonetheless remain crucial to understanding and reproducing the research shown; figures of replicates for experiments for which representative data are shown in the main text can be added here, if brief, or as supplementary data. Mathematical proofs of results not central to the paper can be added as an appendix.

The detailed forms of the Jacobian matrices W, L and V are given by

(A1) $$W=\left[\begin{array}{ccc} -{\hat{r}}_{0,1}^{1}R_{1}^{0}{}^{{}^{T}}y_{0} & 0 & 0\\ R_{1}^{0}{}^{{}^{T}}y_{0} & 0 & 0\\ -R_{2}^{1}{}^{{}^{T}}{\hat{r}}_{0,1}^{1}R_{1}^{0}{}^{{}^{T}}y_{0}-{\hat{r}}_{1,2}^{2}R_{2}^{0}{}^{{}^{T}}y_{0} & -{\hat{r}}_{1,2}^{2}R_{2}^{1}{}^{{}^{T}}y_{0} & 0\\ R_{2}^{0}{}^{{}^{T}}y_{0} & R_{2}^{1}{}^{{}^{T}}y_{0} & 0\\ -R_{3}^{1}{}^{{}^{T}}{\hat{r}}_{0,1}^{1}R_{1}^{0}{}^{{}^{T}}y_{0}-R_{3}^{2}{}^{{}^{T}}{\hat{r}}_{1,2}^{2}R_{2}^{0}{}^{{}^{T}}y_{0}-{\hat{r}}_{2,3}^{3}R_{3}^{0}{}^{{}^{T}}y_{0} & -R_{3}^{2}{}^{{}^{T}}{\hat{r}}_{1,2}^{2}R_{2}^{1}{}^{{}^{T}}y_{0}-{\hat{r}}_{2,3}^{3}R_{3}^{1}{}^{{}^{T}}y_{0} & -{\hat{r}}_{2,3}^{3}R_{3}^{2}{}^{{}^{T}}y_{0}\\ R_{3}^{0}{}^{{}^{T}}y_{0} & R_{3}^{1}{}^{{}^{T}}y_{0} & R_{3}^{2}{}^{{}^{T}}y_{0} \end{array}\right]$$

(A2) $$L=\left[\begin{array}{ccc} \begin{array}{cc} \left(\begin{array}{c} \begin{array}{c} e^{\mu \varphi_{11}}\\ e^{\mu \sum_{j=1}^{2}\varphi_{1j}} \end{array}\\ \begin{array}{c} e^{\mu \sum_{j=1}^{3}\varphi_{1j}}\\ 0_{27\times 1} \end{array} \end{array}\right) & \left(\begin{array}{c} 0_{3\times 1}\\ e^{\mu \varphi_{21}}\\ \begin{array}{c} e^{\mu \sum_{j=1}^{2}\varphi_{2j}}\\ \begin{array}{c} e^{\mu \sum_{j=1}^{3}\varphi_{2j}}\\ 0_{24\times 1} \end{array} \end{array} \end{array}\right) \end{array} & \begin{array}{cc} \left(\begin{array}{c} 0_{6\times 1}\\ e^{\mu \varphi_{31}}\\ \begin{array}{c} e^{\mu \sum_{j=1}^{2}\varphi_{3j}}\\ \begin{array}{c} e^{\mu \sum_{j=1}^{3}\varphi_{3j}}\\ \begin{array}{c} e^{\mu \sum_{j=1}^{4}\varphi_{3j}}\\ e^{\mu \sum_{j=1}^{5}\varphi_{3j}}\\ 0_{19\times 1} \end{array} \end{array} \end{array} \end{array}\right) & \left(\begin{array}{c} 0_{11\times 1}\\ e^{\mu \varphi_{41}}\\ \begin{array}{c} e^{\mu \sum_{j=1}^{2}\varphi_{4j}}\\ \begin{array}{c} e^{\mu \sum_{j=1}^{3}\varphi_{4j}}\\ \begin{array}{c} e^{\mu \sum_{j=1}^{4}\varphi_{4j}}\\ e^{\mu \sum_{j=1}^{5}\varphi_{4j}}\\ 0_{14\times 1} \end{array} \end{array} \end{array} \end{array}\right) \end{array} & \begin{array}{cc} \left(\begin{array}{c} 0_{16\times 1}\\ e^{\mu \varphi_{51}}\\ \begin{array}{c} e^{\mu \sum_{j=1}^{2}\varphi_{5j}}\\ \begin{array}{c} e^{\mu \sum_{j=1}^{3}\varphi_{5j}}\\ \begin{array}{c} e^{\mu \sum_{j=1}^{4}\varphi_{5j}}\\ e^{\mu \sum_{j=1}^{5}\varphi_{5j}}\\ \begin{array}{c} e^{\mu \sum_{j=1}^{6}\varphi_{5j}}\\ e^{\mu \sum_{j=1}^{7}\varphi_{5j}}\\ 0_{7\times 1} \end{array} \end{array} \end{array} \end{array} \end{array}\right) & \left(\begin{array}{c} 0_{23\times 1}\\ e^{\mu \varphi_{61}}\\ \begin{array}{c} e^{\mu \sum_{j=1}^{2}\varphi_{6j}}\\ \begin{array}{c} e^{\mu \sum_{j=1}^{3}\varphi_{6j}}\\ \begin{array}{c} e^{\mu \sum_{j=1}^{4}\varphi_{6j}}\\ e^{\mu \sum_{j=1}^{5}\varphi_{6j}}\\ \begin{array}{c} e^{\mu \sum_{j=1}^{6}\varphi_{6j}}\\ e^{\mu \sum_{j=1}^{7}\varphi_{6j}} \end{array} \end{array} \end{array} \end{array} \end{array}\right) \end{array} \end{array}\right]$$

and

(A3) $$V=\left[\begin{array}{cccccc} 0_{1\times 3} & 0_{1\times 3} & 0_{1\times 3} & 0_{1\times 3} & 0_{1\times 3} & 0_{1\times 3}\\ 0_{1\times 3} & 0_{1\times 3} & 0_{1\times 3} & 0_{1\times 3} & 0_{1\times 3} & 0_{1\times 3}\\ {\bar{l}}_{13}^{1T} & -{\left({\bar{l}}_{13}^{1}\times r_{1,A_{141}}^{1}\right)}^{T} & 0_{1\times 3} & 0_{1\times 3} & 0_{1\times 3} & 0_{1\times 3}\\ 0_{1\times 3} & 0_{1\times 3} & 0_{1\times 3} & 0_{1\times 3} & 0_{1\times 3} & 0_{1\times 3}\\ 0_{1\times 3} & 0_{1\times 3} & 0_{1\times 3} & 0_{1\times 3} & 0_{1\times 3} & 0_{1\times 3}\\ {\bar{l}}_{23}^{1T} & -{\left({\bar{l}}_{23}^{1}\times r_{1,A_{241}}^{1}\right)}^{T} & 0_{1\times 3} & 0_{1\times 3} & 0_{1\times 3} & 0_{1\times 3}\\ 0_{1\times 3} & 0_{1\times 3} & 0_{1\times 3} & 0_{1\times 3} & 0_{1\times 3} & 0_{1\times 3}\\ 0_{1\times 3} & 0_{1\times 3} & 0_{1\times 3} & 0_{1\times 3} & 0_{1\times 3} & 0_{1\times 3}\\ {\bar{l}}_{33}^{1T} & -{\left({\bar{l}}_{33}^{1}\times r_{1,A_{341}}^{1}\right)}^{T} & 0_{1\times 3} & 0_{1\times 3} & 0_{1\times 3} & 0_{1\times 3}\\ 0_{1\times 3} & 0_{1\times 3} & 0_{1\times 3} & 0_{1\times 3} & 0_{1\times 3} & 0_{1\times 3}\\ -{\bar{l}}_{35}^{1T} & {\left({\bar{l}}_{35}^{1}\times r_{1,A_{351}}^{1}\right)}^{T} & {\bar{l}}_{35}^{2T} & -{\left({\bar{l}}_{35}^{2}\times r_{2,A_{362}}^{2}\right)}^{T} & 0_{1\times 3} & 0_{1\times 3}\\ 0_{1\times 3} & 0_{1\times 3} & 0_{1\times 3} & 0_{1\times 3} & 0_{1\times 3} & 0_{1\times 3}\\ 0_{1\times 3} & 0_{1\times 3} & 0_{1\times 3} & 0_{1\times 3} & 0_{1\times 3} & 0_{1\times 3}\\ {\bar{l}}_{43}^{1T} & -{\left({\bar{l}}_{43}^{1}\times r_{1,A_{441}}^{1}\right)}^{T} & 0_{1\times 3} & 0_{1\times 3} & 0_{1\times 3} & 0_{1\times 3}\\ 0_{1\times 3} & 0_{1\times 3} & 0_{1\times 3} & 0_{1\times 3} & 0_{1\times 3} & 0_{1\times 3}\\ -{\bar{l}}_{45}^{1T} & {\left({\bar{l}}_{45}^{1}\times r_{1,A_{451}}^{1}\right)}^{T} & {\bar{l}}_{45}^{2T} & -{\left({\bar{l}}_{45}^{2}\times r_{2,A_{462}}^{2}\right)}^{T} & 0_{1\times 3} & 0_{1\times 3}\\ 0_{1\times 3} & 0_{1\times 3} & 0_{1\times 3} & 0_{1\times 3} & 0_{1\times 3} & 0_{1\times 3}\\ 0_{1\times 3} & 0_{1\times 3} & 0_{1\times 3} & 0_{1\times 3} & 0_{1\times 3} & 0_{1\times 3}\\ {\bar{l}}_{53}^{1T} & -{\left({\bar{l}}_{53}^{1}\times r_{1,A_{541}}^{1}\right)}^{T} & 0_{1\times 3} & 0_{1\times 3} & 0_{1\times 3} & 0_{1\times 3}\\ 0_{1\times 3} & 0_{1\times 3} & 0_{1\times 3} & 0_{1\times 3} & 0_{1\times 3} & 0_{1\times 3}\\ -{\bar{l}}_{55}^{1T} & {\left({\bar{l}}_{55}^{1}\times r_{1,A_{551}}^{1}\right)}^{T} & {\bar{l}}_{55}^{2T} & -{\left({\bar{l}}_{55}^{2}\times r_{2,A_{562}}^{2}\right)}^{T} & 0_{1\times 3} & 0_{1\times 3}\\ 0_{1\times 3} & 0_{1\times 3} & 0_{1\times 3} & 0_{1\times 3} & 0_{1\times 3} & 0_{1\times 3}\\ 0_{1\times 3} & 0_{1\times 3} & -{\bar{l}}_{57}^{2T} & {\left({\bar{l}}_{57}^{2}\times r_{2,A_{572}}^{2}\right)}^{T} & {\bar{l}}_{57}^{3T} & -{\left({\bar{l}}_{57}^{3}\times r_{3,A_{583}}^{3}\right)}^{T}\\ 0_{1\times 3} & 0_{1\times 3} & 0_{1\times 3} & 0_{1\times 3} & 0_{1\times 3} & 0_{1\times 3}\\ 0_{1\times 3} & 0_{1\times 3} & 0_{1\times 3} & 0_{1\times 3} & 0_{1\times 3} & 0_{1\times 3}\\ {\bar{l}}_{63}^{1T} & -{\left({\bar{l}}_{63}^{1}\times r_{1,A_{641}}^{1}\right)}^{T} & 0_{1\times 3} & 0_{1\times 3} & 0_{1\times 3} & 0_{1\times 3}\\ 0_{1\times 3} & 0_{1\times 3} & 0_{1\times 3} & 0_{1\times 3} & 0_{1\times 3} & 0_{1\times 3}\\ -{\bar{l}}_{65}^{1T} & {\left({\bar{l}}_{65}^{1}\times r_{1,A_{651}}^{1}\right)}^{T} & {\bar{l}}_{65}^{2T} & -{\left({\bar{l}}_{65}^{2}\times r_{2,A_{662}}^{2}\right)}^{T} & 0_{1\times 3} & 0_{1\times 3}\\ 0_{1\times 3} & 0_{1\times 3} & 0_{1\times 3} & 0_{1\times 3} & 0_{1\times 3} & 0_{1\times 3}\\ 0_{1\times 3} & 0_{1\times 3} & -{\bar{l}}_{67}^{2T} & {\left({\bar{l}}_{67}^{2}\times r_{2,A_{672}}^{2}\right)}^{T} & {\bar{l}}_{67}^{3T} & -{\left({\bar{l}}_{67}^{3}\times r_{3,A_{683}}^{3}\right)}^{T} \end{array}\right]$$

respectively, where $r_{k_{j},\;A_{ijk_{j}}\;}^{k_{j}}$ denotes the vector from the origin of Frame $O_{k_{j}}$ to the attachment point $A_{ijk_{j}}$ with respect to frame $O_{k_{j}}$; ${\bar{l}}_{ij}^{k_{j}}$ denotes the unit vector of $l_{ij}^{k_{j}}$, which is the cable segment vector from attachment point $A_{ijk_{j}}$ to attachment point $A_{i\left(j+1\right)k_{j+1}}$; the symbol $R_{k^{\prime }}^{k}$ ($k^{\prime },k=0,1,2,3$) denotes the rotation matrix of Frame $O_{k^{\prime }}$ with respect to Frame $O_{k}$; the symbol $y_{0}$ is given as $y_{0}={\left[\begin{array}{ccc} 0 & 1 & 0 \end{array}\right]}^{T}$; and the symbol ˆ is defined as

$$\hat{a}=\left[\begin{array}{ccc} 0 & -a_{3} & a_{2}\\ a_{3} & 0 & -a_{1}\\ -a_{2} & a_{1} & 0 \end{array}\right]$$

for the vector $a={\left(\begin{array}{ccc} a_{1} & a_{2} & a_{3} \end{array}\right)}^{T}$, and $\varphi_{ij}$ denotes the contact angle which can be computed using

(A4) $$\varphi_{ij}=\cos^{-1}\left({\bar{l}}_{i,j-1}^{k_{j}}\cdot {\bar{l}}_{ij}^{k_{j}}\right),$$

where μ denotes the coefficient of saturated viscous friction and is set to 0.2 in the experiment.

## Appendix B

The detailed form of the parameter vector π is given by

(A5) $$\pi ={\left(\begin{array}{ccc} \begin{array}{cc} \begin{array}{cc} \pi_{1} & \pi_{2} \end{array} & \begin{array}{cc} \pi_{3} & \pi_{4} \end{array} \end{array} & \begin{array}{cc} \begin{array}{cc} \pi_{5} & \pi_{6} \end{array} & \begin{array}{cc} \pi_{7} & \pi_{8} \end{array} \end{array} & \begin{array}{cc} \begin{array}{cc} \pi_{9} & \pi_{10} \end{array} & \begin{array}{cc} \pi_{11} & \begin{array}{cc} \pi_{12} & \pi_{13} \end{array} \end{array} \end{array} \end{array}\right)}^{T},$$

where $\pi_{1},\cdots ,\pi_{13}$ can be given by

$\pi_{1}=m_{1},\;\pi_{2}=m_{1}r_{1,C_{1},z}^{1},\pi_{3}=m_{1}r_{1,C_{1},x}^{1},\;\pi_{4}=I_{1,yy}^{1}+m_{1}\left(r_{1,C_{1},z}^{1}{}^{2}+r_{1,C_{1},x}^{1}{}^{2}\right)$,

$\pi_{5}=m_{2},\pi_{6}=m_{2}r_{2,C_{2},z}^{2},\pi_{7}=m_{2}r_{2,C_{2},x}^{2},\pi_{8}=I_{2,yy}^{2}+m_{2}\left(r_{2,C_{2},z}^{2}{}^{2}+r_{2,C_{2},x}^{2}{}^{2}\right)$,

$\pi_{9}=m_{3},\pi_{10}=m_{3}r_{3,C_{3},z}^{3},\pi_{11}=m_{3}r_{3,C_{3},x}^{3},\pi_{12}=I_{3,yy}^{3}+m_{3}\left(r_{3,C_{3},z}^{3}{}^{2}+r_{3,C_{3},x}^{3}{}^{2}\right)$, $\pi_{13}=F_{ii}$.

Here, $F_{ii}$ denotes the i-th diagonal element of F.

The detailed form of the matrix Φ is given by

(A6) $$\Phi =\left(\begin{array}{c} \begin{array}{ccc} \begin{array}{cc} \begin{array}{cc} \phi_{11} & \phi_{12} \end{array} & \begin{array}{cc} \phi_{13} & \phi_{14} \end{array} \end{array} & \begin{array}{cc} \begin{array}{cc} \phi_{15} & \phi_{16} \end{array} & \begin{array}{cc} \phi_{17} & \phi_{18} \end{array} \end{array} & \begin{array}{cc} \begin{array}{cc} \phi_{19} & \phi_{1,10} \end{array} & \begin{array}{cc} \phi_{1,11} & \begin{array}{cc} \phi_{1,12} & \phi_{1,13} \end{array} \end{array} \end{array} \end{array}\\ \begin{array}{ccc} \begin{array}{cc} \begin{array}{cc} \phi_{21} & \phi_{22} \end{array} & \begin{array}{cc} \phi_{23} & \phi_{24} \end{array} \end{array} & \begin{array}{cc} \begin{array}{cc} \phi_{25} & \phi_{26} \end{array} & \begin{array}{cc} \phi_{27} & \phi_{28} \end{array} \end{array} & \begin{array}{cc} \begin{array}{cc} \phi_{29} & \phi_{2,10} \end{array} & \begin{array}{cc} \phi_{2,11} & \begin{array}{cc} \phi_{2,12} & \phi_{2,13} \end{array} \end{array} \end{array} \end{array}\\ \begin{array}{ccc} \begin{array}{cc} \begin{array}{cc} \phi_{31} & \phi_{32} \end{array} & \begin{array}{cc} \phi_{33} & \phi_{34} \end{array} \end{array} & \begin{array}{cc} \begin{array}{cc} \phi_{35} & \phi_{36} \end{array} & \begin{array}{cc} \phi_{37} & \phi_{38} \end{array} \end{array} & \begin{array}{cc} \begin{array}{cc} \phi_{39} & \phi_{3,10} \end{array} & \begin{array}{cc} \phi_{3,11} & \begin{array}{cc} \phi_{3,12} & \phi_{3,13} \end{array} \end{array} \end{array} \end{array} \end{array}\right),$$

where $\phi_{11},\cdots ,\phi_{1,13}$, $\phi_{21},\cdots ,\phi_{2,13}$ and $\phi_{31},\cdots ,\phi_{3,13}$ can be given by

$$\begin{array}{c} \phi_{11}=G_{1}{\ddot{\theta }}_{1,r}-\;gd_{1},\phi_{12}=2r_{0,1,z}^{1}{\ddot{\theta }}_{1,r}-gs_{1\alpha_{11}\alpha_{12}},\phi_{13}=2r_{0,1,x}^{1}{\ddot{\theta }}_{1,r}-gc_{1\alpha_{11}\alpha_{12}},\phi_{14}={\ddot{\theta }}_{1,r},\\ \phi_{15}=\left(2D_{1}+G_{1}+G_{2}\right){\ddot{\theta }}_{1,r}+\left(D_{1}+G_{2}\right){\ddot{\theta }}_{2,r}+D_{1}^{’}{\dot{\theta }}_{2}{\dot{\theta }}_{2,r}+D_{1}^{’}\left({\dot{\theta }}_{2}{\dot{\theta }}_{1,r}+{\dot{\theta }}_{1}{\dot{\theta }}_{2,r}\right)-g\left(d_{1}+d_{2}\right),\\ \phi_{16}=2\left(a_{2}+r_{1,2,z}^{2}\right){\ddot{\theta }}_{1,r}+\left(a_{2}+2r_{1,2,z}^{2}\right){\ddot{\theta }}_{2,r}+b_{2}{\dot{\theta }}_{2}{\dot{\theta }}_{2,r}+b_{2}\left({\dot{\theta }}_{2}{\dot{\theta }}_{1,r}+{\dot{\theta }}_{1}{\dot{\theta }}_{2,r}\right)-gs_{2\alpha_{21}\alpha_{22}1\alpha_{11}\alpha_{12}},\\ \phi_{17}=2\left(b_{2}+r_{1,2,x}^{2}\right){\ddot{\theta }}_{1,r}+\left(b_{2}+2r_{1,2,x}^{2}\right){\ddot{\theta }}_{2,r}-a_{2}{\dot{\theta }}_{2}{\dot{\theta }}_{2,r}-a_{2}\left({\dot{\theta }}_{2}{\dot{\theta }}_{1,r}+{\dot{\theta }}_{1}{\dot{\theta }}_{2,r}\right)-gc_{2\alpha_{21}\alpha_{22}1\alpha_{11}\alpha_{12}},\\ \phi_{18}={\ddot{\theta }}_{1,r}+{\ddot{\theta }}_{2,r},\\ \phi_{19}=\left(2D_{1}+2D_{2}+2D_{3}+G_{1}+G_{2}+G_{3}\right){\ddot{\theta }}_{1,r}+\left(D_{1}+2D_{2}+D_{3}+G_{2}+G_{3}\right){\ddot{\theta }}_{2,r}+\left(D_{2}+D_{3}+G_{3}\right){\ddot{\theta }}_{3,r}+\left(D_{1}^{’}+D_{3}^{’}\right){\dot{\theta }}_{2}{\dot{\theta }}_{2,r}\\ +\left(D_{2}^{’}+D_{3}^{’}\right){\dot{\theta }}_{3}{\dot{\theta }}_{3,r}+\left(D_{1}^{’}+D_{3}^{’}\right)\left({\dot{\theta }}_{2}{\dot{\theta }}_{1,r}+{\dot{\theta }}_{1}{\dot{\theta }}_{2,r}\right)+\left(D_{2}^{’}+D_{3}^{’}\right)\left({\dot{\theta }}_{3}{\dot{\theta }}_{1,r}+{\dot{\theta }}_{1}{\dot{\theta }}_{3,r}\right)+\left(2D_{2}^{’}+D_{3}^{’}\right){\dot{\theta }}_{3}{\dot{\theta }}_{2,r}+D_{3}^{’}{\dot{\theta }}_{2}{\dot{\theta }}_{3,r}-g\left(d_{1}+d_{2}+d_{3}\right),\\ \phi_{1,10}=2\left(a_{3}+a_{4}+r_{2,3,z}^{3}\right){\ddot{\theta }}_{1,r}+\left(2a_{3}+a_{4}+2r_{2,3,z}^{3}\right){\ddot{\theta }}_{2,r}+\left(a_{3}+a_{4}+2r_{2,3,z}^{3}\right){\ddot{\theta }}_{3,r}+b_{4}{\dot{\theta }}_{2}{\dot{\theta }}_{2,r}+\left(b_{3}+b_{4}\right){\dot{\theta }}_{3}{\dot{\theta }}_{3,r}\\ +b_{4}\left({\dot{\theta }}_{2}{\dot{\theta }}_{1,r}+{\dot{\theta }}_{1}{\dot{\theta }}_{2,r}\right)+\left(b_{3}+b_{4}\right)\left({\dot{\theta }}_{3}{\dot{\theta }}_{1,r}+{\dot{\theta }}_{1}{\dot{\theta }}_{3,r}\right)+b_{4}{\dot{\theta }}_{2}{\dot{\theta }}_{3,r}+\left(2b_{3}+b_{4}\right){\dot{\theta }}_{3}{\dot{\theta }}_{2,r}-gs_{3\alpha_{31}\alpha_{32}2\alpha_{21}\alpha_{22}1\alpha_{11}\alpha_{12}},\\ \phi_{1,11}=2\left(b_{3}+b_{4}+r_{2,3,x}^{3}\right){\ddot{\theta }}_{1,r}+\left(2b_{3}+b_{4}+2r_{2,3,x}^{3}\right){\ddot{\theta }}_{2,r}+\left(b_{3}+b_{4}+2r_{2,3,x}^{3}\right){\ddot{\theta }}_{3,r}-a_{4}{\dot{\theta }}_{2}{\dot{\theta }}_{2,r}-\left(a_{3}+a_{4}\right){\dot{\theta }}_{3}{\dot{\theta }}_{3,r}\\ -a_{4}\left({\dot{\theta }}_{2}{\dot{\theta }}_{1,r}+{\dot{\theta }}_{1}{\dot{\theta }}_{2,r}\right)-\left(a_{3}+a_{4}\right)\left({\dot{\theta }}_{3}{\dot{\theta }}_{1,r}+{\dot{\theta }}_{1}{\dot{\theta }}_{3,r}\right)-a_{4}{\dot{\theta }}_{2}{\dot{\theta }}_{3,r}-\left(2a_{3}+a_{4}\right){\dot{\theta }}_{3}{\dot{\theta }}_{2,r}-gc_{3\alpha_{31}\alpha_{32}2\alpha_{21}\alpha_{22}1\alpha_{11}\alpha_{12}},\\ \phi_{1,12}={\ddot{\theta }}_{1,r}+{\ddot{\theta }}_{2,r}+{\ddot{\theta }}_{3,r},\phi_{1,13}={\dot{\theta }}_{1,r}.\\ \phi_{21}=0,\phi_{22}=0,\phi_{23}=0,\phi_{24}=0,\phi_{25}=\left(D_{1}+G_{2}\right){\ddot{\theta }}_{1,r}+G_{2}{\ddot{\theta }}_{2,r}-D_{1}^{’}{\dot{\theta }}_{1}{\dot{\theta }}_{1,r}-gd_{2},\\ \phi_{26}=\left(a_{2}+2r_{1,2,z}^{2}\right){\ddot{\theta }}_{1,r}+2r_{1,2,z}^{2}{\ddot{\theta }}_{2,r}-b_{2}{\dot{\theta }}_{1}{\dot{\theta }}_{1,r}-gs_{2\alpha_{21}\alpha_{22}1\alpha_{11}\alpha_{12}},\\ \phi_{27}=\left(b_{2}+2r_{1,2,x}^{2}\right){\ddot{\theta }}_{1,r}+2r_{1,2,x}^{2}{\ddot{\theta }}_{2,r}+a_{2}{\dot{\theta }}_{1}{\dot{\theta }}_{1,r}-gc_{2\alpha_{21}\alpha_{22}1\alpha_{11}\alpha_{12}},\\ \phi_{28}={\ddot{\theta }}_{1,r}+{\ddot{\theta }}_{2,r},\\ \phi_{29}=\left(D_{1}+2D_{2}+D_{3}+G_{2}+G_{3}\right){\ddot{\theta }}_{1,r}+\left(2D_{2}+G_{2}+G_{3}\right){\ddot{\theta }}_{2,r}+\left(D_{2}+G_{3}\right){\ddot{\theta }}_{3,r}-\left(D_{1}^{’}+D_{3}^{’}\right){\dot{\theta }}_{1}{\dot{\theta }}_{1,r}+D_{2}^{’}{\dot{\theta }}_{3}{\dot{\theta }}_{3,r}+2D_{2}^{’}{\dot{\theta }}_{1}{\dot{\theta }}_{3,r}\\ +D_{2}^{’}\left({\dot{\theta }}_{2}{\dot{\theta }}_{3,r}+{\dot{\theta }}_{3}{\dot{\theta }}_{2,r}\right)-g\left(d_{2}+d_{3}\right),\\ \phi_{2,10}=\left(2a_{3}+a_{4}+2r_{2,3,z}^{3}\right){\ddot{\theta }}_{1,r}+2\left(a_{3}+r_{2,3,z}^{3}\right){\ddot{\theta }}_{2,r}+\left(a_{3}+2r_{2,3,z}^{3}\right){\ddot{\theta }}_{3,r}-b_{4}{\dot{\theta }}_{1}{\dot{\theta }}_{1,r}+b_{3}{\dot{\theta }}_{3}{\dot{\theta }}_{3,r}+2b_{3}{\dot{\theta }}_{1}{\dot{\theta }}_{3,r}\\ +b_{3}\left({\dot{\theta }}_{2}{\dot{\theta }}_{3,r}+{\dot{\theta }}_{3}{\dot{\theta }}_{2,r}\right)-gs_{3\alpha_{31}\alpha_{32}2\alpha_{21}\alpha_{22}1\alpha_{11}\alpha_{12}},\\ \phi_{2,11}=\left(2b_{3}+b_{4}+2r_{2,3,x}^{3}\right){\ddot{\theta }}_{1,r}+2\left(b_{3}+r_{2,3,x}^{3}\right){\ddot{\theta }}_{2,r}+\left(b_{3}+2r_{2,3,x}^{3}\right){\ddot{\theta }}_{3,r}+a_{4}{\dot{\theta }}_{1}{\dot{\theta }}_{1,r}-a_{3}{\dot{\theta }}_{3}{\dot{\theta }}_{3,r}-2a_{3}{\dot{\theta }}_{1}{\dot{\theta }}_{3,r}\\ -a_{3}\left({\dot{\theta }}_{2}{\dot{\theta }}_{3,r}+{\dot{\theta }}_{3}{\dot{\theta }}_{2,r}\right)-gc_{3\alpha_{31}\alpha_{32}2\alpha_{21}\alpha_{22}1\alpha_{11}\alpha_{12}},\\ \phi_{2,12}={\ddot{\theta }}_{1,r}+{\ddot{\theta }}_{2,r}+{\ddot{\theta }}_{3,r},\;\phi_{2,13}={\dot{\theta }}_{2,r}.\\ \phi_{31}=0,\phi_{32}=0,\phi_{33}=0,\phi_{34}=0,\phi_{35}=0,\phi_{36}=0,\phi_{37}=0,\phi_{38}=0,\\ \phi_{39}=\left(D_{2}+D_{3}+G_{3}\right){\ddot{\theta }}_{1,r}+\left(D_{2}+G_{3}\right){\ddot{\theta }}_{2,r}+G_{3}{\ddot{\theta }}_{3,r}-\left(D_{2}^{’}+D_{3}^{’}\right){\dot{\theta }}_{1}{\dot{\theta }}_{1,r}-D_{2}^{’}{\dot{\theta }}_{2}{\dot{\theta }}_{2,r}-2D_{2}^{’}{\dot{\theta }}_{1}{\dot{\theta }}_{2,r}-gd_{3},\\ \phi_{3,10}=\left(a_{3}+a_{4}+2r_{2,3,z}^{3}\right){\ddot{\theta }}_{1,r}+\left(a_{3}+2r_{2,3,z}^{3}\right){\ddot{\theta }}_{2,r}+2r_{2,3,z}^{3}{\ddot{\theta }}_{3,r}-\left(b_{3}+b_{4}\right){\dot{\theta }}_{1}{\dot{\theta }}_{1,r}-b_{3}{\dot{\theta }}_{2}{\dot{\theta }}_{2,r}-2b_{3}{\dot{\theta }}_{1}{\dot{\theta }}_{2,r}-gs_{3\alpha_{31}\alpha_{32}2\alpha_{21}\alpha_{22}1\alpha_{11}\alpha_{12}},\\ \phi_{3,11}=\left(b_{3}+b_{4}+2r_{2,3,x}^{3}\right){\ddot{\theta }}_{1,r}+\left(b_{3}+2r_{2,3,x}^{3}\right){\ddot{\theta }}_{2,r}+2r_{2,3,x}^{3}{\ddot{\theta }}_{3,r}+\left(a_{3}+a_{4}\right){\dot{\theta }}_{1}{\dot{\theta }}_{1,r}+a_{3}{\dot{\theta }}_{2}{\dot{\theta }}_{2,r}+2a_{3}{\dot{\theta }}_{1}{\dot{\theta }}_{2,r}-gc_{3\alpha_{31}\alpha_{32}2\alpha_{21}\alpha_{22}1\alpha_{11}\alpha_{12}},\\ \phi_{3,12}={\ddot{\theta }}_{1,r}+{\ddot{\theta }}_{2,r}+{\ddot{\theta }}_{3,r},\phi_{3,13}={\dot{\theta }}_{3,r}. \end{array}$$

$c_{.}\equiv cos\left(\cdot \right),\;s_{.}\equiv sin\left(\cdot \right)$, for example, $c_{2\alpha_{21}\alpha_{22}}=cos\left(\theta_{2}+\alpha_{21}+\alpha_{22}\right)$ and others are similar, the symbols $\alpha_{21}$, $\alpha_{22}$, $\alpha_{31}$, $\alpha_{32}$, $\alpha_{41}$ and $\alpha_{42}$ denote the parameters of the links and are all constants and the symbol $I_{k,yy}^{k}$ denotes the y component of the inertia tensor relative to the center of mass of Link k with respect to the current frame. In Equation (A6), there are

$$\begin{array}{c} D_{1}=r_{0,1,z}^{1}r_{1,2,z}^{2}+r_{0,1,x}^{1}r_{1,2,x}^{2},\;D_{2}=r_{1,2,z}^{2}r_{2,3,z}^{3}+r_{1,2,x}^{2}r_{2,3,x}^{3},\;D_{3}=r_{0,1,z}^{1}r_{2,3,z}^{3}+r_{0,1,x}^{1}r_{2,3,x}^{3},\\ E_{1}=r_{0,1,x}^{1}r_{1,2,z}^{2}-r_{0,1,z}^{1}r_{1,2,x}^{2},\;E_{2}=r_{1,2,x}^{2}r_{2,3,z}^{3}-r_{1,2,z}^{2}r_{2,3,x}^{3},\;E_{3}=r_{0,1,x}^{1}r_{2,3,z}^{3}-r_{0,1,z}^{1}r_{2,3,x}^{3},\\ G_{1}=r_{0,1,z}^{1}{}^{2}+r_{0,1,x}^{1}{}^{2},G_{2}=r_{1,2,z}^{2}{}^{2}+r_{1,2,x}^{2}{}^{2},G_{3}=r_{2,3,z}^{3}{}^{2}+r_{2,3,x}^{3}{}^{2},a_{2}=r_{0,1,z}^{1}c_{2\alpha_{21}\alpha_{22}}+r_{0,1,x}^{1}s_{2\alpha_{21}\alpha_{22}},\\ a_{3}=r_{1,2,z}^{2}c_{3\alpha_{31}\alpha_{32}}+r_{1,2,x}^{2}s_{3\alpha_{31}\alpha_{32}},\;a_{4}=r_{0,1,z}^{1}c_{3\alpha_{31}\alpha_{32}2\alpha_{21}\alpha_{22}}+r_{0,1,x}^{1}s_{3\alpha_{31}\alpha_{32}2\alpha_{21}\alpha_{22}},\\ b_{2}=r_{0,1,x}^{1}c_{2\alpha_{21}\alpha_{22}}-r_{0,1,z}^{1}s_{2\alpha_{21}\alpha_{22}},\;b_{3}=r_{1,2,x}^{2}c_{3\alpha_{31}\alpha_{32}}-r_{1,2,z}^{2}s_{3\alpha_{31}\alpha_{32}},\\ b_{4}=r_{0,1,x}^{1}c_{3\alpha_{31}\alpha_{32}2\alpha_{21}\alpha_{22}}-r_{0,1,z}^{1}s_{3\alpha_{31}\alpha_{32}2\alpha_{21}\alpha_{22}},d_{1}=r_{0,1,x}^{1}c_{1\alpha_{11}\alpha_{12}}+r_{0,1,z}^{1}s_{1\alpha_{11}\alpha_{12}},\\ d_{2}=r_{1,2,x}^{2}c_{2\alpha_{21}\alpha_{22}1\alpha_{11}\alpha_{12}}+r_{1,2,z}^{2}s_{2\alpha_{21}\alpha_{22}1\alpha_{11}\alpha_{12}},\\ d_{3}=r_{2,3,x}^{3}c_{3\alpha_{31}\alpha_{32}2\alpha_{21}\alpha_{22}1\alpha_{11}\alpha_{12}}+r_{2,3,z}^{3}s_{3\alpha_{31}\alpha_{32}2\alpha_{21}\alpha_{22}1\alpha_{11}\alpha_{12}},\\ D_{1}=D_{1}c_{2\alpha_{21}\alpha_{22}}+E_{1}s_{2\alpha_{21}\alpha_{22}},\;D_{2}=D_{2}c_{3\alpha_{31}\alpha_{32}}+E_{2}s_{3\alpha_{31}\alpha_{32}},\;D_{3}=D_{3}c_{3\alpha_{31}\alpha_{32}2\alpha_{21}\alpha_{22}}+E_{3}s_{3\alpha_{31}\alpha_{32}2\alpha_{21}\alpha_{22}},\\ D_{1}^{’}=E_{1}c_{2\alpha_{21}\alpha_{22}}-D_{1}s_{2\alpha_{21}\alpha_{22}},D_{2}^{’}=E_{2}c_{3\alpha_{31}\alpha_{32}}-D_{2}s_{3\alpha_{31}\alpha_{32}},D_{3}^{’}=E_{3}c_{3\alpha_{31}\alpha_{32}2\alpha_{21}\alpha_{22}}-D_{3}s_{3\alpha_{31}\alpha_{32}2\alpha_{21}\alpha_{22}}. \end{array}$$
